# Amyloid Precursor-Like Protein 2 deletion-induced retinal synaptopathy related to congenital stationary night blindness: structural, functional and molecular characteristics

**DOI:** 10.1186/s13041-016-0245-z

**Published:** 2016-06-08

**Authors:** Virginie Dinet, Giuseppe D. Ciccotosto, Kimberley Delaunay, Céline Borras, Isabelle Ranchon-Cole, Corinne Kostic, Michèle Savoldelli, Mohamed El Sanharawi, Laurent Jonet, Caroline Pirou, Na An, Marc Abitbol, Yvan Arsenijevic, Francine Behar-Cohen, Roberto Cappai, Frédéric Mascarelli

**Affiliations:** Centre de Recherche des Cordeliers, Université Paris Descartes, Université Pierre et Marie Curie, Paris, France; Department of Pathology and Bio21 Molecular Science and Biotechnology Institute, The University of Melbourne, Melbourne, Australia; Laboratoire de Biophysique Sensorielle, Université Clermont 1, Clermont-Ferrand, France; Unit of Gene Therapy & Stem Cell Biology, University of Lausanne, Jules-Gonin Eye Hospital, Lausanne, Switzerland

**Keywords:** Amyloid precursor protein, Amyloid precursor-like protein 2, Synapses, Synaptopathy, Differentiation, Congenital stationary night blindness, Transcription

## Abstract

**Background:**

Amyloid precursor protein knockout mice (APP-KO) have impaired differentiation of amacrine and horizontal cells. APP is part of a gene family and its paralogue amyloid precursor-like protein 2 (APLP2) has both shared as well as distinct expression patterns to APP, including in the retina. Given the impact of APP in the retina we investigated how APLP2 expression affected the retina using APLP2 knockout mice (APLP2-KO).

**Results:**

Using histology, morphometric analysis with noninvasive imaging technique and electron microscopy, we showed that APLP2-KO retina displayed abnormal formation of the outer synaptic layer, accompanied with greatly impaired photoreceptor ribbon synapses in adults. Moreover, APLP2-KO displayed a significant decease in ON-bipolar, rod bipolar and type 2 OFF-cone bipolar cells (36, 21 and 63 %, respectively). Reduction of the number of bipolar cells was accompanied with disrupted dendrites, reduced expression of metabotropic glutamate receptor 6 at the dendritic tips and alteration of axon terminals in the OFF laminae of the inner plexiform layer. In contrast, the APP-KO photoreceptor ribbon synapses and bipolar cells were intact. The APLP2-KO retina displayed numerous phenotypic similarities with the congenital stationary night blindness, a non-progressive retinal degeneration disease characterized by the loss of night vision. The pathological phenotypes in the APLP2-KO mouse correlated to altered transcription of genes involved in pre- and postsynatic structure/function, including CACNA1F, GRM6, TRMP1 and Gα0, and a normal scotopic a-wave electroretinogram amplitude, markedly reduced scotopic electroretinogram b-wave and modestly reduced photopic cone response. This confirmed the impaired function of the photoreceptor ribbon synapses and retinal bipolar cells, as is also observed in congenital stationary night blindness. Since congenital stationary night blindness present at birth, we extended our analysis to retinal differentiation and showed impaired differentiation of different bipolar cell subtypes and an altered temporal sequence of development from OFF to ON laminae in the inner plexiform layer. This was associated with the altered expression patterns of bipolar cell generation and differentiation factors, including MATH3, CHX10, VSX1 and OTX2.

**Conclusions:**

These findings demonstrate that APLP2 couples retina development and synaptic genes and present the first evidence that APLP2 expression may be linked to synaptic disease.

**Electronic supplementary material:**

The online version of this article (doi:10.1186/s13041-016-0245-z) contains supplementary material, which is available to authorized users.

## Background

The amyloid precursor protein (APP) is known to play, together with its proteolytic fragments, numerous and varied roles in cell physiology and pathology, including in Alzheimer disease [1–4]. The amyloid precursor-like protein 2 (APLP2) belongs to the APP gene family. Phylogenetic analysis suggests the functions of APP, APLP1 and APLP2 have diverged after duplication to mediate distinct neuronal actions [5]. Our knowledge of the physiological and pathophysiological functions of APLP2 remains poor relative to APP functions, and no neuronal pathology has been definitively associated with APLP2. In contrast, APLP2 has recently been identified as one of the myopia genes and APLP2-KO mice develop high degrees of hyperopia [6]. APLP2 expression patterns during brain development suggest an important role in maturation of specific neuronal subtypes in the central nervous system (CNS) [7–9]. However, the expression or role of APLP2 in the developing and adult retina, and whether there is functional redundancy between APLP2 and APP in the retina, is not known.

As part of the CNS, the neural retina is arranged in well-defined laminar structures. Photoreceptors (rods and cones) transmit visual information to ganglion cells via bipolar cells. In the “classical” rod pathway, rods feed into rod bipolar cells that provide input to both the ON and OFF pathway via AII/glycinergic amacrine cells. The rod bipolar cell light-evoked response is initiated by a decrease in glutamate occupancy of mGluR6, which decreases its G-protein activity culminating in the opening of the TRPM1 channel [10–12]. The mGluR6-mediated modulation of TRPM1 also is known to require several other proteins including Gα_o_ and GRP179 [12–16] and other known components with unresolved function including Nyctalopin and LRIT3 [17]. Most of these proteins are localized at the dendritic tips of ON-bipolar cells. In adult mice, APP is expressed in horizontal, bipolar, amacrine and ganglion cells and on the two retinal synaptic layers: inner plexiform layer (IPL) and outer plexiform layer (OPL) [18, 19]. The IPL contains bipolar-ganglion cell connections and modulatory amacrine conventional synapses. We showed that APP is necessary for the differentiation of AII amacrine cells [18]. The OPL contains the ribbon synapses between photoreceptor cells and the bipolar and horizontal interneurons. Defects at these synapses led to congenital stationary night blindness (CSNB).

CSNB encompasses a group of genetically and clinically heterogeneous retinal disorders that primarily affect night vision. The non-progressive night vision loss is present at birth and there is no cure for CSNB. The prevalence of CSNB is approximately 1 in 10,000 [20]. CSNB is caused by mutations in seventeen identified genes with an unknown number yet to be identified and inherited in an autosomal dominant, autosomal recessive or X-linked recessive manner [21–23]. Clinically, CSNB is classified as the Riggs type and the Schubert-Bornschein type based on specific waveforms on the electroretinogram (ERG). The rare Riggs type is characterized by decreased scotopic a-wave amplitude in keeping with rod photoreceptor dysfunction and by normal cone system [24]. This should be distinguished from the Schubert-Bornschein type of ERG abnormalities in which scotopic a-wave amplitude is normal [25]. The Schubert-Bornschein type is divided into two sub-types, complete CSNB (cCSNB or CSNB1), associated with a drastically reduced or completely abolished scotopic b-wave response, because of a failure to transmit the photoreceptor signal through the ON bipolar cells, and incomplete CSNB (iCSNB or CSNB2), which is associated with a reduced scotopic b-wave and reduced photopic cone responses indicating ON- and OFF-bipolar cell dysfunction [26]. cCNSB has been associated with mutations in genes that encode postsynaptic proteins which are required for the signal transduction cascade responsible for bipolar cell depolarization, including *GRM6*, *TRPM1*, *GRP179*, *NYX* and *LRIT3* [27–33]. Mutations in *CACNA1F* were identified in patients with iCNSB [34, 35]. *CACNA1F* encodes the α1F subunit of the L-type voltage-gated Ca^2+^ channel, Ca_v_1.4, and is located at the photoreceptor ribbon synapse. Abnormal synapses in the outer nuclear layer (ONL) detected by a noninvasive imaging technique using optical coherence tomography (OCT) have been observed in some cases of iCSNB [36], while thinning of the ganglion cell layer (GCL), IPL and inner nuclear layer (INL) in other iCSNB cases [37]. Mutations in *CABP4*, encoding a Ca^2+^-binding protein located in photoreceptor synaptic terminals and associated with Ca_v_1.4, lead to autosomal recessive iCSNB [38]. Most of the cases associated with *CABP4* mutations have recently been shown to display high hyperopia [39, 40]. A minority of iCSNB patients have impaired night vision compared to cCSNB [41] and they have less severely impaired night vision and have a more variable phenotype with respect to the visual acuity, refractive error (myopia/hyperopia) and the b/a wave amplitude ratios of the scotopic ERG than those with cCSNB [41]. In mouse models of iCSNB, both hypo- and hyperactivated channels accomplished respectively by deletion of *CABP4* and the Cacna1f I745T mutation lead to similar ERG alterations, visual impairments and an improper maturation of the synapse architecture [42, 43], indicating that impaired retinal synaptogenesis may contribute to vision impairment in iCSNB.

Although different genes responsible for the pathogenic mechanisms of CSNB have been identified, further studies are needed to clarify the molecular mechanisms of the disease. For CSNB patients in whom the genetic causes are still to be discovered, mutations are likely to be found in genes that function in photoreceptor pre- and postsynaptic processes that affect retinal transmission. It was suggested that *BHLHB4*, *MATH5*, or *PRDM8*, which encode three transcription factors required for either maturation of rod bipolar cells or differentiation of several bipolar cell subtypes, may be candidate genes for CSNB [44, 45]. Recently, the differentiation factor of rod bipolar cells, PRDM8, was presented as a candidate gene for CSNB [46], reinforcing the importance to study the mechanisms of bipolar cell differentiation for elucidating the pathological events involved in CSNB. A canine pedigree of cCSNB where the ON-bipolar cell function is compromised has been recently characterized [47]. In this study all known CSNB genes have been excluded, suggesting the existence of still to be identified genes involved in the disease process.

Given the role of APLP2 in synapse function in adult brain and peripheral nervous system (PNS) [3, 48], and the effect of APP on the differentiation of AII amacrine cells [18], we investigated the role of APLP2 in synaptic lamination in the adult retina using APLP2-KO mice. APLP2-KO mice display no signs of retinal degeneration in young and adult. In this study, the lack of APLP2 expression resulted in numerous phenotypic similarities to CSNB. APLP2-KO retina had a nonprogressive reduction of the scotopic ERG b-wave and the photopic cone responses. This was associated with an affected OPL structure comprising impaired presynaptic photoreceptor ribbon complex and a postsynaptic element of ON and OFF-bipolar cells resembling iCSNB. We describe for the first time the role of APLP2 in neuronal differentiation which involves regulating the temporal expression of a combination of intrinsic factors implicated in the control of bipolar cell generation (early postnatal) and differentiation (late postnatal) during development, and in pre and postsynatic structure/function in adults. APLP2 may be a link between retina development and synaptic genes involved in the pathogenic events in CSNB.

## Results

### Laminar expression of APLP2 in postnatal developing and adult retinas

**Fig. 1:**
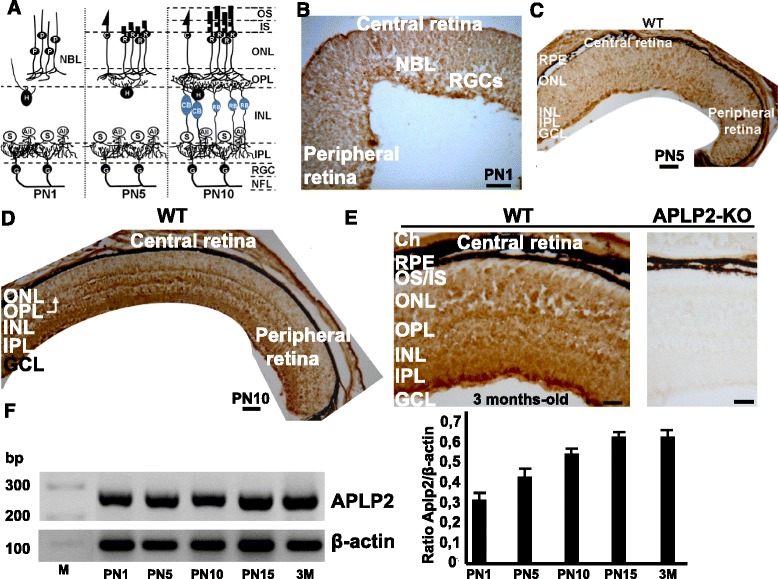
APLP2 protein and mRNA expression in postnatal developing mouse retinas. **a** Schema of developmental sequence in the mouse retina. Longitudinal cryostat sections of WT (**b**-**e**) and APLP2-KO (**e**) retinas were immunostained with anti-APLP2 antibody at early postnatal (PN) developmental stages; (**b**) PN1, (**c**) PN5, and (**d**) PN10 and in the adult (**e**) 3 months-old (3 M). APLP2 was expressed through the different waves of retinal differentiation. G: ganglion cells, AII: AII amacrine cells, S: starburst amacrine cells, P: photoreceptors, H: horizontal cells, R: rods, C: cones, CB: cone bipolar cells, RB: rod bipolar cells, NBL: neuroblastic retina layer, RGCs: retinal ganglion cells, GCL: ganglion cell layer, INL: inner nuclear layer, ONL: outer nuclear layer, IPL: inner plexiform layer, OPL: outer plexiform layer, IS: inner segment, OS: outer segment, RPE: retinal pigment epithelium, E: embryonic stage, PN: postnatal stage. Scale bar 50 μm. (F) Total RNA extracted from WT retinas at the different ages indicated. APLP2 and β-actin mRNA levels visualized by ethidium bromide staining and quantified by RT-PCR. mRNA levels for APLP2 are standardized to β-actin content from the same RNA sample. Means ± s.e.m., *N* = 5 mice per age group

Developmental expression of APLP2 was examined by immunohistochemistry on WT neural retinas. During postnatal development, retinal ganglion cells (RGCs) and amacrine cells are the first cells to differentiate and form the IPL (Fig. 1a). Then, horizontal cells and photoreceptors differentiate and contact each other in the outer retina, forming the OPL. Later, the vertical networks are interconnected when bipolar cells are generated and connections with ganglion cells are established. At postnatal day (PN) 1, strong APLP2 immunostaining was detected in the ganglion cell layer (GCL), which harbours RGCs, whereas weaker but uniform APLP2 immunostaining was observed in the neuroblastic layer (NBL) (Fig. 1b). At PN5, the APLP2 immunostaining remained strong in the GCL and INL, where differentiating amacrine and bipolar cells are located (Fig. 1c). At PN10, the GCL, INL and OPL, which contains the presynaptic ribbon complex of the photoreceptors and the postsynaptic plexus of the bipolar cells, were all intensely stained (Fig. 1d). However, in the IPL, which is composed of conventional synapses between RGCs and amacrine and bipolar cells, APLP2 immunostaining was heterogeneous. In adult retina, the GCL, IPL, INL and OPL immunostaining for APLP2 remained strong, whereas APLP2 immunostaining was weak in the photoreceptor layer (Fig. 1e, left panel). Control experiments with the anri-APLP2 antibody on retinal sections of APLP2-KO showed no APLP2 immunostaining (Fig. 1e, right panel). RT-PCR analysis showed that APLP2 mRNA levels from whole retina increased during the retinal differentiation period from PN1 to PN15, then plateauing at adult age (Fig. 1f).

Using a panel of antibodies against specific markers of retinal cell subtypes, we showed that APLP2 colocalized with RGCs, amacrine (starburst and AII), rod bipolar and horizontal cells in adult retina (Additional file 1: Figure S1A-F). In the IPL, APLP2 colocalized with the three calretinin-labeled laminae including the upper and lower laminae which correspond to the OFF and ON starburst cell plexuses respectively (Additional file 1: Figure S1C) and with the two strata S2 and S4 bands of the cholinergic synapses (Additional file 1: Figure S1D). In the OPL, APLP2 colocalized with VGLUT1, the only vesicular glutamate transporter located in the ribbon synapses of photoreceptors (Additional file 1: Figure S1F) and with PSD95, located presynaptically at photoreceptor synapses (cone pedicles and rod spherules) (Additional file 1: Figure S1G). Moreover, co-immunostaining with anti-APLP2 and anti-APP antibodies showed that APLP2 colocalized with APP in all synaptic and nuclear layers of neural retina (Additional file 1: Figure S1I-M).

### Deletion of APLP2 alters the structure of the OPL

**Fig. 2:**
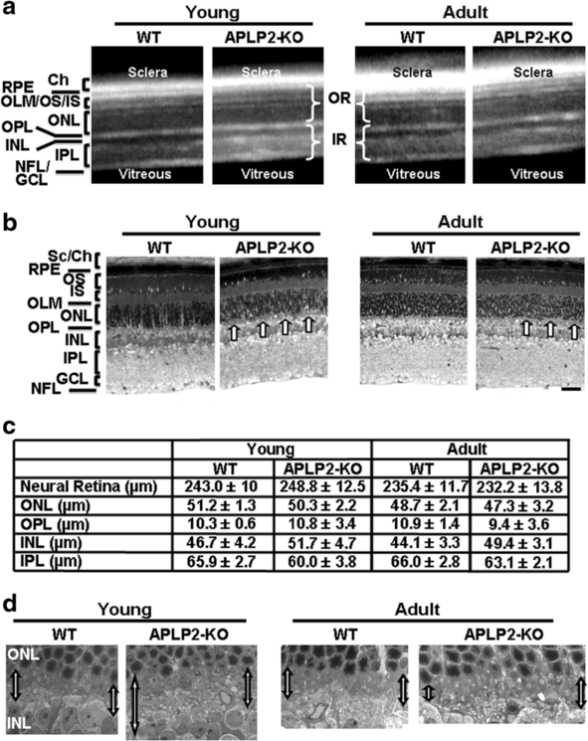
The laminar structure of the retina was similar between WT and APLP2-KO except for the OPL. **a** SD-OCT sections revealed no gross alteration of the young and adult retinas. **b** Ex vivo histology of 80 nm-thick resin retina sections showed an irregular OPL (arrows) in the young and adult APLP2-KO. **c** Thicknesses of major retinal layers in WT and APLP2-KO young and adult retinas determined using Visilog 6.4 version software (Noesis). Mean ± s.e.m., *N* = 4 mice per genotype. **d** Electron micrographs showed an irregular thickness and pathological aspect of the OPL in young and adult APLP2-KO

We investigated retinal structure in APLP2-KO by spectral domain optical coherence tomography (SD-OCT), histology and transmission electron microscopy (TEM) in young and adult mice. The SD-OCT images showed an identical laminar organization in the inner and outer retina in young APLP2-KO and WT (Fig. 2a) mice since the thicknesses of the total neural retina, inner retina (inclusive of GCL, IPL and INL) and outer retina (inclusive of OPL, ONL, OLM/IS/OS) were not significantly different (Additional file 1: Table S2). Semithin histological sections confirmed that the gross laminar structure of the retina was similar between the two genotypes (Fig. 2b). In contrast, the profile of the OPL was irregular in APLP2-KO as compared to its uniform thickness in WT (Fig. 2b, arrows). CNSB and X-linked retinoschisis (XRLS) are two main retinal disorders specifically affecting the OPL. Both disorders show altered ERG b-wave, but XLRS is progressive [49–51], while CSNB is stationary. Moreover, XLRS is characterized by a cystic degeneration of the retina, leading to a split of retinal layers and frequently accompanied with retinal pigment epithelium (RPE) atrophy and retinal detachment during adulthood. SD-OCT images (Fig. 2a) showed that overall, retinal morphology did not change over time. No focal lesions from the retinal pigment epithelium (RPE)/Bruch’s membrane complex to the GCL/NFL were observed in adult mice. No retinal detachments were observed (Fig. 2b). At high magnification (6200-8700x magnification), TEM of the RPE, in association with the photoreceptor outer segments, showed normally developed RPE with elongated apical processes into the interphotoreceptor space for WT and APLP2-KO (Additional file 1: Figure S2 A-D). The presence of phagosomes, newly engulfed by the RPE, is predicted to be the product of functional light-induced phagocytosis of the distal tip of the photoreceptor outer segment by the RPE cells. The TEM micrographs of longitudinal sections confirmed the absence of RPE atrophy and retinal detachment in adult APLP2-KO. RPE flatmounts visualized by phalloidin staining showed a uniform hexagonal array of the F-actin cytoskeletal network in WT and APLP2-KO (Additional file 1: Figure S2 E and F). Taken together, these data show a well-structured RPE/choroid complex in APLP2-KO mice.

The thicknesses of the total neural retina, inner retina and outer retina were not significantly different between young and adult APLP2-KO (Additional file 1: Table S2). The gross laminar structure of the retina was similar between the two genotypes at all ages examined (Fig. 2b). Morphometric analysis of semithin histological sections showed a comparable thickness of the neural retina in both groups of age of APLP2-KO (Fig. 2c) confirming SD-OCT data, while the photoreceptor cell nuclei number was similar between the two genotypes in both groups of age (Additional file 1: Table S2). We saw no differences between WT and APLP2-KO retinas when comparing the number of cones stained for cone arrestin (Additional file 1: Table S2). The average OPL thicknesses were not different between WT and APLP2-KO, although width of OPL was irregular in APLP2-KO (Fig. 2b arrows and c). High-magnification (1650 x magnification) TEM micrographs of longitudinal sections confirmed the irregular width in the OPL of the APLP2-KO compared to WT for both groups of age (Fig. 2d). Taken together, these data indicate that loss of APLP2 leads to a discrete stationary structural alteration of the OPL thickness.

### Impaired photoreceptor-bipolar cell synaptic transmission in APLP2-KO

**Fig. 3:**
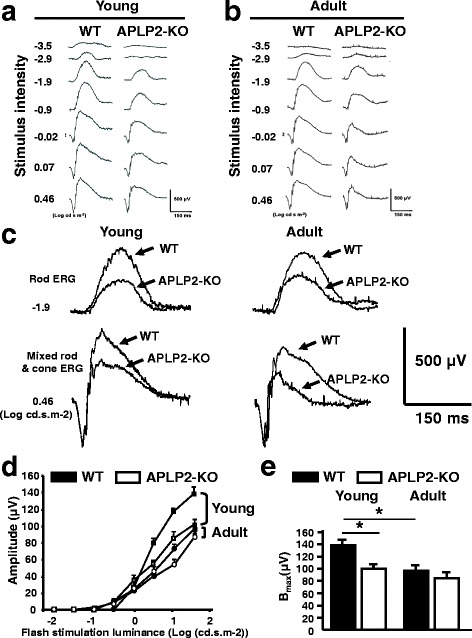
APLP2-KO mice have a nonprogressive defect in retinal responses postsynaptic to rod photoreceptors and cone circuitry function. Representative voltage traces from flash ERG recording in young (**a**) and adult (**b**) dark-adapted WT (left column) and APLP2-KO (right column) mice. Numbers on left of the traces correspond to the luminances (log cd.s.m^−2^) of the flashes that elicit these responses. The scale indicates 150 ms and 500 μV. **c** Examples of comparisons of responses obtained from WT and APLP2-KO mice are illustrated by superimposing the respective traces obtained from these two mouse genotypes. Responses to low-intensity stimuli (−1.9 log cd.s.m^−2^ under dark adaptation) and responses to high-stimuli (0.46 log cd.s.m^−2^ under dark adaptation). **d** Amplitude of the cone ERG b-wave in young (scare symbol) and adult (round symbol) APLP2-KO (white symbols) and WT (black symbols). **e** Maximal b-wave amplitude (*B*_*max*_) in young and adult APLP2-KO (white bars) and WT (black bars). Means ± s.e.m., *N* = 6 mice per genotype. *N* = 6 mice per age group. *, *p* < 0.05. (*t-test*)

Due to the similarities between APLP2-KO mice and CSNB in the phenotypic features of the OPL, we hypothesized APLP2-KO mice have dysfunction in the transmission pathway from photoreceptors to bipolar cells. Therefore, we tested our hypothesis by assessing retinal function under scotopic conditions, using ERG in APLP2-KO. Figure 3a shows a series of representative ERGs of young WT and APLP2-KO mice recorded under dark-adapted conditions to flash stimuli that cover a 4-log-unit range of intensity. The scotopic ERG of WT mice shows a typical a- and b-wave pattern. At all intensities, the ERG included a positive-polarity b-wave, which represent the summed activity of rod bipolar cells, while at the higher intensities, the ERG also included a negative a-wave that is generated by the light-induced closure of cGMP-gated ion channels along the rod photoreceptor outer segments. Under a dim light (low flash intensity: −3.5 log cdsm^−2^), a b-wave with a subnormal amplitude was observed in young APLP2-KO (Fig. 3a and Additional file 1: Figure S3A). Under intermediate and bright flash intensities, the b-wave was markedly reduced in amplitude in APLP2-KO compared to WT. At the higher intensities, the a-wave of young APLP2-KO appeared normal. Superimposing the ERGs recorded at a flash intensity of −1.9 log cdsm^−2^ and 0.46 log cdsm^−2^ evoking a rod response and a combined rod-cone response, respectively, highlighted the difference in b-wave amplitude in young APLP2-KO and WT (Fig. 3c). The average data at all flash intensities show that the ERG a-wave was similar in young APLP2-KO and WT in amplitude (Additional file 1: Figure S3A) and implicit time (Additional file 1: Figure S3C). In contrast, the ERG b-wave amplitude was reduced in young APLP2-KO (Additional file 1: Figure S3A). However, the b-wave implicit time was not altered in APLP2-KO compared to WT (Additional file 1: Figure S3C). The maximal b-wave amplitude was significantly (*p* < 0.05) reduced in APLP2-KO (866 ± 191 μV) compared to WT (1218 ± 115 μV) with no alteration in half saturation luminance (*K*) or the slope of the b-wave sensitivity curve (*n*) (Additional file 1: Figure S3E). When b-wave amplitudes were normalized by calculating the b/a-wave ratio, we observed that values from young APLP2-KO were significantly lower at the brightest flash intensity (b/a: 2.9 vs. 1.9 in WT vs. APLP2-KO, respectively). These data indicate the lack of APLP2 affects the amplification gain of synaptic transmission from the photoreceptors to the bipolar cells, whereas the phototransduction in rod photoreceptors is not affected. To establish whether ERG deficits of APLP2-KO were stationary or progressive, we repeated the scotopic ERGs in adults. Figure 3b shows a series of representative ERGs of adult WT and APLP2-KO mice. Superimposing the ERGs recorded at a flash intensity of −1.9 log cdsm^−2^ and 0.46 log cdsm^−2^ highlighted the difference in b-wave amplitude in adult APLP2-KO and WT (Fig. 3c). The average data show that the ERG-a wave of adult APLP2-KO was comparable with WT (Additional file 1: Figure S3B and D), indicating that photoreceptor functions remained normal in APLP2-KO overtime. Therefore, we excluded APLP2-KO as a model of the Riggs type. ERG b-wave responses of APLP2-KO declined in amplitude at the same rate in adult and young compared to WT of the same age (compare Additional file 1: Figure S3A with Additional file 1: Figure S3B), with no alteration in the *K* and *n* parameters (Fig. 3e). In adults, the maximal b-wave amplitude was significantly (p < 0.05) reduced in APLP2-KO (772 ± 155 μV) compared to WT (1135 ± 211 μV) (Additional file 1: Figure S3E). At the brightest flash intensity, the b/a-wave ratio was significantly reduced in adult APLP2-KO compared to adult WT (b/a: 2.5 vs. 1.9 in WT vs. APLP2-KO, respectively), while similar in young and adult APLP2-KO. Therefore, we hypothesized that APLP2-KO resemble the incomplete form of the Schubert-Bornschein type of CSNB.

The cone photopic ERG is moderately altered in iCSNB patients, while markedly reduced in cCSNB [52, 53]. Therefore, we investigated the cone circuitry function under photopic (light adapted) conditions to confirm APLP2-KO as a model of iCSNB. Because photopic a-waves are negligible in mice [54], the amplitude and implicit time of the photopic b-waves were measured. The cone-mediated responses were recorded at a range of light intensities (−1.5 log to 1.5 log cds/m^−2^). With increasing flash intensities, the b-wave amplitude increased incrementally in both age groups but the maximal cone b-wave amplitude was significantly (*p* < 0.05) reduced in adult (97.1 ± 8.9 μV) compared to young WT (138.5 ± 8.5 μV) (Fig. 3d), consistent with age-related declines in cone function in C57BL/6 mice [55, 56]. In young, the maximal b-wave amplitude was significantly reduced (*p* < 0.05) in APLP2-KO (100.3 ± 7.8 μV) compared to WT (138.5 ± 8.5 μV) (Fig. 3d), indicating cone-mediated pathway dysfunction in young APLP2-KO. However, the b-wave implicit time was not altered in APLP2-KO compared to WT (data not shown). Moreover, the maximal amplitude of the cone ERG b-wave were comparable in both age groups for APLP2-KO mice (103 ± 7.8 μV vs. 87.7 ± 11.4 μV in young vs. adult, respectively) (Fig. 3e), indicating a non-progressive phenotype of APLP2-KO for cone dysfunction. Altogether these data indicate that loss of APLP2 in young and adult mice leads to an electrophysiological defect in retinal circuitry from the photoreceptors (rods and cones) to the bipolar cells resembling the iCSNB.

### APLP2 is necessary for proper presynaptic ribbon and postsynaptic triad morphology of adult OPL

**Fig. 4:**
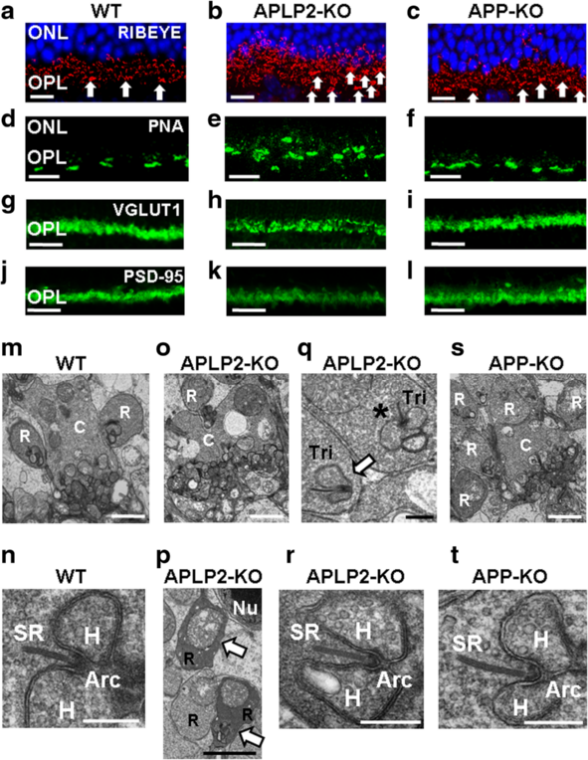
Morphology, location and ultrastructure of the ribbon photoreceptor presynaptic terminals in the adult retina differed dramatically between WT, APLP2-KO and APP-KO. **a**-**l** Longitudinal cryostat sections of retinas immunostained for (**a**-**c**) RIBEYE, (**d**-**f**) PNA, (**g**-**i**) VGLUT1 and (**j**-**l**) PSD-95 from (**a**, **d**, **g** and **j**) WT, (**b**, **e**, **h** and **k**) APLP2-KO and (**c**, **f**, **i** and **l**) APP-KO mice. **a**-**c** Arrows indicate cone pedicles. **m**-**t** Electron micrographs of longitudinal sections of (**m** and **n**) WT, (**o**-**r**) APLP2-KO and (**s** and **t**) APP-KO. Arrows indicate rod spherules which displayed a densely stained retracting morphology (**p**) and triads laterally oriented (**q**), instead of facing the OPL (asterisk). Cone terminal (C), rod terminal (R), synaptic ribbon (SR), Tri (triad), Arciform density (Arc). Scale bars: 15 μm (**a**-**c**); 20 μm (**d**-**l**); 2 μm (**m**,**o**,**p**,**s**); 0.5 μm (**n**,**q**,**r**,**t**)

Ca_v_1.4-KO mice show altered localization in the OPL of presynaptic proteins, including RIBEYE and PSD95, and impaired distribution of PNA-labeled cone pedicles [57]. Further characterization of the OPL alteration in adult APLP2-KO mice was initially achieved using confocal microscopy and presynaptic marker antibodies to identify the photoreceptors. Four to six rows of RIBEYE-labeled rod ribbon and one row of clustering of RIBEYE at cone synaptic terminals (pedicles) were present in WT (Fig. 4a). In contrast, up to ten misaligned rows of disorganized rod ribbon synapses were detected in APLP2-KO OPL, suggesting the laminar restriction of rod presynaptic terminals (spherules) was compromised (Fig. 4b). The majority of RIBEYE-labeled ribbons showed non-accurate and punctuate-shaped structures, indicating signs of collapse of photoreceptor ribbons. Moreover, ectopic localization of RIBEYE-labeled structures was readily detected in large and numerous foci in the ONL (Fig. 4b). The laminar organization of the pedicles was also altered in APLP2-KO (Fig. 4b, arrows). While the RIBEYE staining in the APP-KO OPL was similar to WT, faint loci of RIBEYE-stained photoreceptor ribbon were observed deep within the ONL in APP-KO (Fig. 4c). In WT retina, one row of faint, aligned and regularly spaced horizontal (fluorescent-conjugated peanut agglutinin) PNA-stained cone pedicles was detected in the OPL (Fig. 4d). In contrast, PNA staining in APLP2-KO retina revealed the laminar restriction of cone pedicles was compromised (Fig. 4e). The PNA staining in APP-KO was similar to WT (Fig. 4f). We also monitored the expression of VGLUT1. VGLUT1 immunostaining was regular and dense in the OPL of WT and APP-KO (Fig. 4g and i). In contrast, APLP2-KO OPL had irregular VGLUT1 immunostaining (Fig. 4h). PSD-95 is used as a common marker for the photoreceptor presynaptic terminals [58] and has an atypical distribution in ribbon synapses. We observed a regular immunostaining pattern for PSD-95 in the OPL of WT and APP-KO (Fig. 4j and l). However, PSD-95 immunostaining was diffuse in the APLP2-KO OPL (Fig. 4k).

We next used TEM (16 K - 41 K x magnification) to monitor the photoreceptor terminals at the ultrastructure level. In the WT OPL region, the cone pedicles contained multiple triads composed of two lateral horizontal processes flanking a central bipolar cell dendrite (Fig. 4m). The majority of the rod spherules contained a unique long presynaptic ribbon and a visible arciform density at its base (Fig. 4n). In APLP2-KO, cone pedicles were irregular in shape and completely disorganized (Fig. 4o). Many rod spherules also displayed a densely stained retracting morphology (Fig. 4p, arrows). Moreover, many triads were misaligned and turned such that the presynaptic ribbon and the two lateral horizontal cells were laterally oriented (Fig. 4q, arrow), instead of facing the OPL (Fig. 4q, asterisk). At higher magnification (64 K x magnification), TEM of the few well-organized APLP2-KO synapses showed a unique long ribbon and a visible arciform density at its base (Fig. 4r), akin to WT synapses (Fig. 4n). In APP-KO, although the cones pedicles were not as well organized as in WT, they contained multiple well-developed triads (Fig. 4s). Higher magnification showed that the rod spherules still had a normal overall photoreceptor terminal architecture (Fig. 4t). Similar alterations of the presynaptic ribbon synapse and postsynaptic triad morphology, including disorganized rod spherules with misoriented synaptic ribbons (Additional file 1: Figure S4C and E, arrows), and irregular cone pedicles (Additional file 1: Figure S4D), were observed in young APLP2-KO.

### APLP2 is necessary for the proper generation of bipolar cells and their dendritic branching and not horizontal cells

**Fig. 5:**
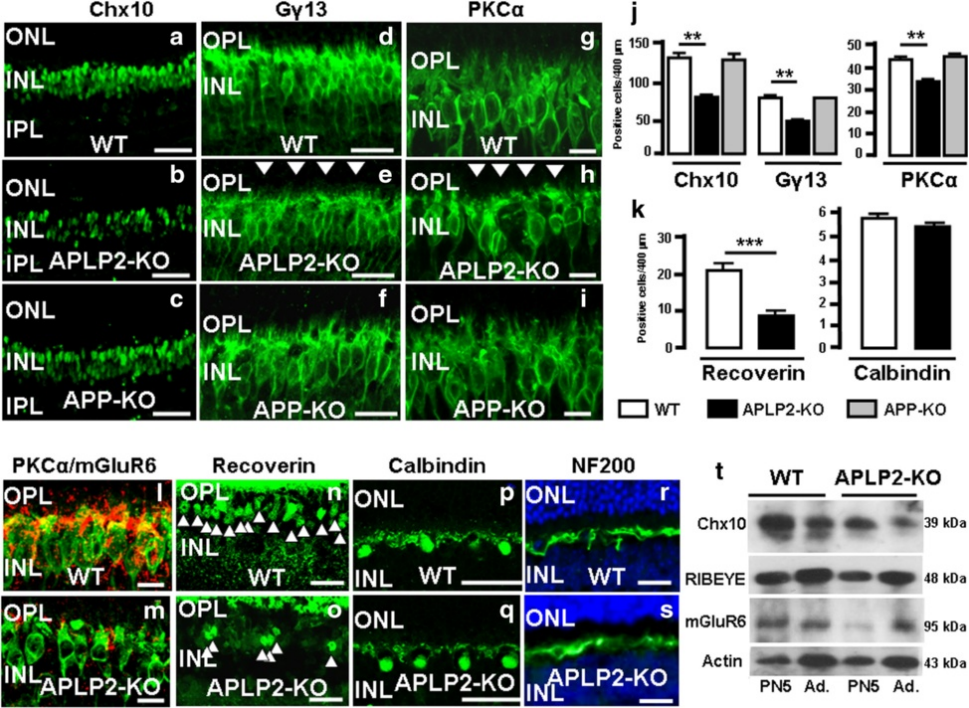
The distribution, organization and number of bipolar cells in the APLP2-KO adult retina differ to WT and APP-KO. Longitudinal cryostat sections of retina were stained for (**a**-**c**) Chx10, (**d**-**f**) Gγ_13_ and (**g**-**i**) PKCα positive bipolar cells. The dendritic branches appeared diffuse and in some cases cells lacked processes on their outer surface (**e** and **h**, arrowheads). **l** and **m** mGluR6 (red) has an abnormal staining pattern of bipolar cell processes verified by double staining with PKCα (green). **n** and **o** Recoverin stains for Type 2 OFF cone bipolar cells in the INL region (arrowheads). Longitudinal cryostat sections of retinas were immunostained for (**p** and **q**) calbindin and (**r** and **s**) NF200 and counterstained with DAPI (blue). Immunostained sections from WT (**a**, **d**, **g**, **l**, **n**, **p** and **r**), APLP2-KO (**b**, **e**, **h**, **m**, **o**, **q** and **s**) and APP-KO (**c**, **f** and **i**) retinas were compared. Scale bars: 80 μm (**a**-**c**); 40 μm (**d**-**f**); 15 μm (**g**, **h**, **i**, **l** and **m**); 50 μm (**n** and **o**); 70 μm (**p** and **q**); 10 μm (**r** and **s**). Quantification of (**j**) bipolar cells and (**k**) type 2 OFF cone bipolar and horizontal cells in WT, APLP2-KO and APP-KO retina performed by counting number of positive nuclei per 400 μm field. Mean ± s.e.m., *N* = 3 mice per genotype. **, *p* < 0.01; ***, *p* < 0.005. (*t-test*). (**t**) Western blot analysis of WT and APLP2-KO protein extracts of the PN5 and adult retinas were probed for Chx10 (39 kDa), RIBEYE (48 kDa), mGluR6 (95 kDa) and the loading control, actin (43 kDa)

Next, we investigated alterations of the postsynaptic bipolar and horizontal cells in adult retinas by immunohistochemistry. Anti-Chx10 antibody, which specifically labels all the bipolar cells in adult retina, revealed large stained Chx10 nuclei of bipolar cells in the INL of all genotypes (Fig. 5a-c). Quantifying the number Chx10-positive cells identified a significant decrease (41.6 %) in APLP2-KO compared to WT, with no change in APP-KO (Fig. 5j). Gγ_13,_ a specific marker for ON bipolar cells (rod bipolar and cone bipolar cells (types 5a and 5b and types 6–9)) stained three rows of ON bipolar cell somas in the WT INL (Fig. 5d). In comparison, APLP2-KO had only one or two rows of ON bipolar cell somas (Fig. 5e) and the number of Gγ_13_-positive ON bipolar cells was significantly reduced (36.4 %) in the INL (Fig. 5j). In addition, the APLP2-KO Gγ_13_ labeled dendritic branches appeared diffuse and in some cases the cells appeared without processes on their outer surface (Fig. 5e, arrowheads). In contrast, the ON bipolar cell soma row structure, cell number and profuse dendritic branching was similar between APP-KO and WT retina (Fig. 5d, f and j). Rod bipolar cells, which were identified by PKCα staining, displayed a regular laminar structure in WT retina (Fig. 5g), while APLP2-KO rod bipolar cell somas were less structured and had a disorderly arrangement (Fig. 5h). There was a significant reduction (21.4 %) in the number of PKCα–positive rod bipolar cells in APLP2-KO compared to WT (Fig. 5j) and the dendritic branches were also less profuse (Fig. 5h, arrowheads). Unlike APLP2-KO, the rod bipolar cell soma structure, content and dendritic arborization was similar between APP-KO and WT (Fig. 5g, i and j). Since the rod bipolar cells utilize a metabotropic pathway to sense light-induced variations in photoreceptor glutamate release, we double immunostained for the metabotropic glutamate receptor mGluR6 and PKCα in the retina. The mGluR6 co-localized to the dendritic tips of rod bipolar cells in WT retina, but in the APLP2-KO retina, the mGluR6 staining was substantially diminished (Fig. 5l and m respectively). We also found differences in the distribution and number of recoverin-positive type 2 OFF-cone bipolar cell somas between WT INL, which appeared in a regular manner, while in the APLP2-KO INL, their were significantly fewer positively stained cells (63.4 % reduction; Fig. 5n and o, arrowheads, k). In contrast to bipolar cells, we saw no differences between WT and APLP2-KO retinas when comparing the number and morphology of horizontal cell somas stained for calbindin (Fig. 5k, p and q respectively) or in the morphology of the horizontal cell axons in the OPL region when staining with anti-neurofilament 200 (Fig. 5r and s). APLP2-KO retinas showed altered synapses with either mislocation (e.g. RIBEYE) or reduced expression (e.g. mGluR6) of synaptic components associated with reduced number of Chx10-positive bipolar cells. Therefore, we performed Western blotting that compares the levels of Chx10 and the two synaptic markers RIBEYE and mGluR6 in WT and APLP2 KO differentiated retinas (Fig. 5t). Western blot analysis showed reduced levels of Chx10 in protein extracts of PN5 and adult APLP2-KO mice compared with WT mice and thus confirms the microscopy and cell counting data showing reduced numbers of bipolar cells in APLP2 compared with WT mice (Fig. 5t and Additional file 1: Table S2). We observed reduced levels of mGLUR6 and increased levels of RIBEYE in adult APLP2-KO compared with WT and thus confirm the microscopy observations. Moreover, we observed reduced levels of mGluR6 and increased levels of RIBEYE levels in PN5 APLP2-KO compared with WT (Fig. 5t and Additional file 1: Table S2), suggesting synaptic alteration during postnatal development in APLP2-KO retinas.

### APLP2 is required for the differentiation of bipolar cells during post-natal development through the correct combination of differentiation transcription factors

CSNB is present at birth. It was recently suggested that impaired development of photoreceptor synapses associated with defect in synaptic transmission contributes to vision impairment in two mouse models of iCSNB [42]. Therefore, we hypothesized APLP2-KO have defects in the development of the OPL. First, we examined APLP2-KO retinas during development. Histological examination of APLP2-KO retinas from E14 to PN1 showed a normal structural organization of the retinas (Additional file 1: Figure S5 A-C). In WT PN5, the photoreceptors initiate ribbon synapse formation [59] and the OPL begins to form (Additional file 1: Figure S5D), however in APLP2-KO the OPL remained undefined (Additional file 1: Figure S5E).

**Fig. 6:**
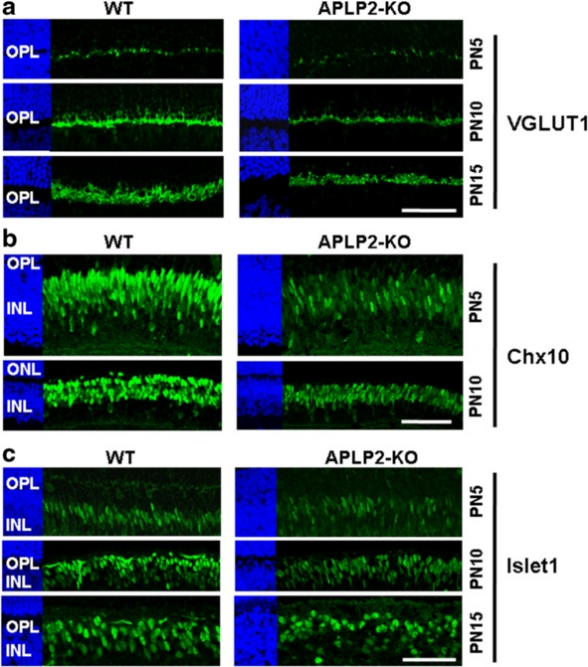
Bipolar and amacrine cell defects in APLP2-KO retina during postnatal development. Longitudinal cryostat sections of retinas from indicated times (PN5, PN10 or PN15) immunostained for (**a**) VGLUT1, (**b**) Chx10, (**c**) Islet1, Scale bars: 50 μm (**a**-**c**)

Then, we investigated whether the observed delay in the development of the APLP2-KO OPL may be secondary to abnormal cell differentiation using markers for postnatal retina differentiation. We initially looked at VGLUT1 expression because it provides a distinct temporal sequence ordering between cones and rods in developing mouse OPL [59]. In WT retina at PN5, VGLUT1 was first expressed with the onset of synaptogenesis in cone pedicles, when they invaded the OPL, yet fewer VGLUT1-positive cone terminals were observed in APLP2-KO (Fig. 6a, upper panel). At PN10, intense VGLUT1 immunostaining was observed in WT retina when the rod spherules invaded the OPL, whereas VGLUT1 immunostaining was weak and thin in APLP2-KO (Fig. 6a, middle panel). By PN15, the mouse retina is fully differentiated into its three nuclear layers and two plexiform layers. The OPL in PN15 WT retina showed a bright and intense band of VGLUT1-positive cone and rod terminals, whereas VGLUT1 immunostaining was weak and diffuse in APLP2-KO (Fig. 6a, lower panel). The observed delay in postnatal development of photoreceptor presynaptic terminals in the APLP2-KO retina was confirmed by the significant decrease in the brightness intensity level quantitated for VGLUT1 immunostaining at each PN5, PN10 and PN15 age group examined (Additional file 1: Table S3). Moreover, we investigated defects in the differentiation of bipolar cells during retinal development. Chx10 is the earliest known pan-bipolar cell marker and its levels peak during bipolar differentiation and persist through adulthood [60, 61]. At PN5 and PN10, Chx10 immunostaining levels in APLP2-KO were weaker in the INL (Fig. 6b), and the number of Chx10-positive nuclei of bipolar cells was significantly decreased by 55.1 and 33.4 % at PN5 and PN10 respectively as compared to WT (Additional file 1: Table S3). This was consistent with findings in the Western blot analysis (Fig. 5t). Islet1 expression was also examined since it is important for differentiation of bipolar cells [62]. At PN5, PN10 and PN15, the number of Islet1-postive bipolar cells in the middle portion of the APLP2-KO INL was significantly reduced by 59.7, 36.0 and 29.7 %, respectively when compared to WT (Fig. 6c and Additional file 1: Table S3). This supports the model that APLP2 participates in bipolar cell differentiation. Then, we investigated the expression of photoreceptor cell and bipolar cell markers during postnatal development by Western blotting. (Additional file 1: Figure S5F). WT and APLP2-KO protein extracts of the PN5 retinas were probed for VGLUT1 and Islet1. We observed a reduction in the levels of VGLUT1 and Islet1 in the APLP2-KO retinas as compared with WT retinas. Quantification of the amounts of each protein from the Western blot confirmed the overall reduction of the bipolar cell and photoreceptor terminal markers in PN5 APLP2-KO retinas (Additional file 1: Table S3), which was consistent with findings in the immunohistochemistry assay.

**Fig. 7:**
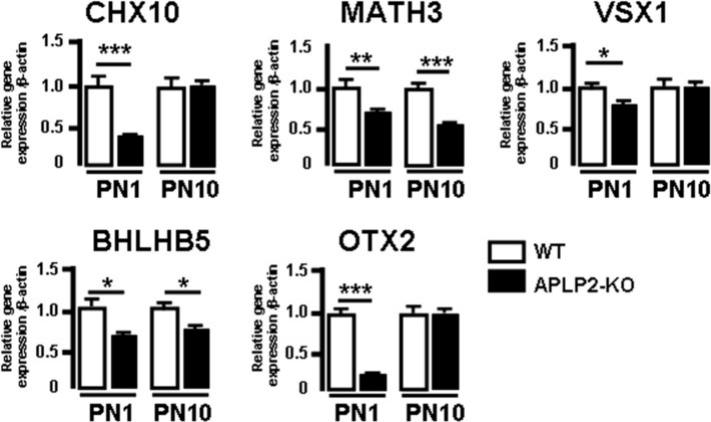
APLP2 is required for correct combination of differentiation transcription factors of bipolar cells. Quantification of differentially expressed retinal genes coding for transcription factors involved in the generation and differentiation of bipolar cells at developmental stages PN1 and PN10 for WT and APLP2-KO by qRT-PCR. Mean ± s.e.m of 3 mice per age group for each genotype showing the gene expression level relative to β-actin. *, *p* < 0.05; **, *p* < 0.01; ***, *p* < 0.005. (*t-test*)

To elucidate the molecular mechanisms underlying bipolar cell defects in APLP2-KO retinal development we analyzed at PN1 and PN10 the mRNA levels of the major basic helix-loop-helix (bHLH) (MATH3 and BHLHB5) and homeodomain (HD) (CHX10, VSX1 and OTX2) factors involved in the generation or differentiation of bipolar cells [63–67]. Using qRT-PCR we found that at PN1 CHX10 and MATH3, the two main factors that promote bipolar cell generation, were significantly downregulated in APLP2-KO compared with WT (Fig. 7). The three major transcription factors involved in the differentiation of bipolar cell subtypes, VSX1 (OFF cone bipolar cells) and BHLHB5 (type 2 OFF cone bipolar cells) and OTX2 (rod bipolar cells), were also significantly downregulated (Fig. 7). Interestingly at PN10 the HD factors CHX10, VSX1 and OTX2 were unchanged, while in contrast the bHLH factors MATH3 and BHLHB5 remained significantly downregulated in APLP2-KO (Fig. 7). Taken together, these data demonstrate that deletion of APLP2 induced impaired regulation of the temporal expression of a combination of intrinsic factors involved in the control of bipolar cell generation (early postnatal) and differentiation (late postnatal) during development.

### APLP2 is necessary for the development of bipolar cell dendritic tips and the generation of the ON and OFF pathways through the proper development of bipolar cell terminals

**Fig. 8:**
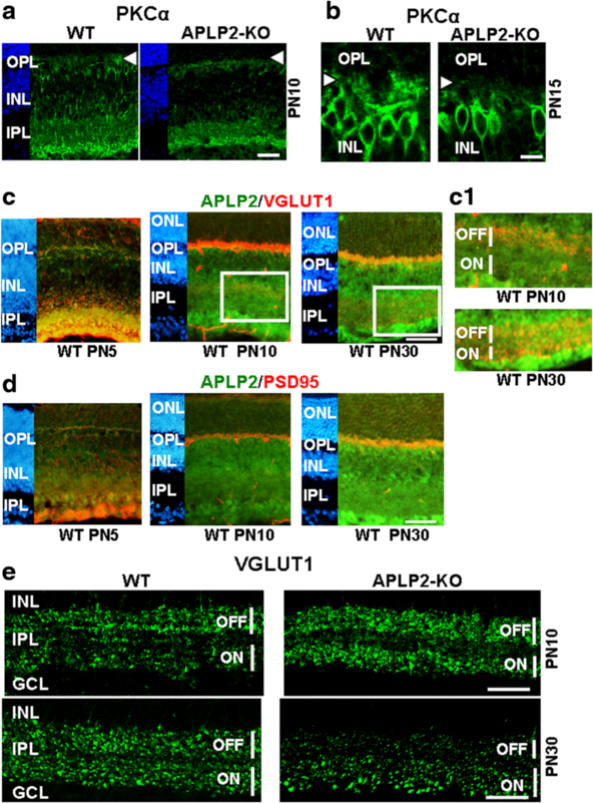
APLP2 is required for the differentiation of bipolar cells and synaptogenesis of cone and rod terminals during postnatal development. Longitudinal cryostat sections of retinas from the indicated stages of postnatal development were immunostained for (**a** and **b**) PKCα, (**c**, C1 and **e**) VGLUT1 and (**d**) PSD95. C1 shows magnified IPL from PN10 and PN30 retinas. Retina sections were double immunostained for APLP2 (green) and VGLUT1 (red) (**c** and C1), and for APLP2 (green) and PSD95 (red) (**d**). Expression of VGLUT1 was analyzed to investigate temporal ordering in development of rod versus cone terminals in the OPL. 50 μm (**a**, **c** and **e**) and 20 μm (**b**)

Since rod bipolar cell dendrites contact photoreceptor terminals in the OPL by PN10, we investigated PKCα immunostaining of bipolar cell dendrites at this developmental stage. In WT, PKCα-labeled rod bipolar cells displayed their typical elongated cell somas in the INL and developed dendrites (Fig. 8a, arrowhead). In contrast, APLP2-KO had fewer rod bipolar cell somas were observed and the dendritic branches were non-existent (Fig. 8a, arrowhead). At PN15, the APLP2-KO dendritic branches were diffuse and in some cases cells lacked processes on their outer surface as compared to WT (Fig. 8b, arrowhead). Altogether, these data reveal a role for APLP2 in the differentiation of bipolar cells and development of their dendritic tips during postnatal development.

Expression of VGLUT1 in bipolar cell terminals presents a temporal sequence of expression from OFF to ON laminae during the differentiation of the IPL, in addition to be expressed in the ribbon synapses of photoreceptors in the OPL [60]. Therefore, we investigated APLP2 and VGLUT1 colocalization during postnatal development and in differentiated WT retinas (Fig. 8c and C1). At PN5, VGLUT1 and APLP2 staining mainly colocalized in the IPL, indicating that APLP2 labels rod bipolar cell terminals and ON cone bipolar cell terminals (Fig. 8c). APLP2 weakly stained the OPL and no colocalization of APLP2 and VGLUT1 was observed because VGLUT1 is not expressed in the OPL at this developmental stage (Fig. 8c). At PN10, VGLUT1 and APLP2 staining colocalized in the OPL of WT mice (Fig. 8c). Moreover, staining for VGLUT1 was preferentially located in the OFF sublamina where it colocalized with APLP2 staining (Fig. 8c and C1, magnified region from PN10 IPL). This data confirms localization of APLP2 in bipolar cell terminals during postnatal development. In differentiated retinas (PN30), VGLUT1 and APLP2 still colocalized presynaptically in the OPL (Fig. 8c). VGLUT1 showed equal staining intensity in the bipolar cell terminals of the OFF and ON sublamina, and APLP2 and VGLUT1 colocalized in the two sublaminas (Fig. 8c and C1, magnified region from PN30 IPL). At PN5, PSD95 was mainly observed in the IPL (Fig. 8d). APLP2 and PSD95 colocalized at bipolar cell ribbon synapses (Fig. 8d), consistent with the postsynaptic localization of PSD95 in the IPL. At this stage, a weak PSD95 and APLP2 co-staining was observed in the OPL. Immunofluorescence double staining of APLP2 and PSD95 confirms localization of APLP2 in ribbon synapses of photoreceptor cells from PN10 to adult WT OPL (Fig. 8d).

APLP2 is strongly expressed in the two retinal synaptic layers, IPL and OPL, during postnatal development, and we observed a delay in the development of the APLP2-KO OPL. Therefore, we investigated VGLUT1 expression in the IPL to study the differentiation of the bipolar cell synapse, during postnatal development in APLP2-KO. In WT, discrete VGLUT1 immunostaining was first detected in the inner retina at PN5, whereas no VGLUT1 immunostaining was observed in APLP2-KO (data not shown). At PN10, WT staining for VGLUT1 was preferentially located in the OFF sublamina where the bipolar cell terminals were located (Fig. 8e). In PN10 APLP2-KO there was more intense VGLUT1 immunostaining and an abnormal stratification pattern with two strong VGLUT1-stained sublaminae separated from each other by a large and weakly stained VGLUT1 band (Fig. 8e). Quantitative analysis of the confocal images in the OFF and ON sublaminae showed a significant increase of VGLUT1 immunostaining in APLP2-KO compared to WT (Additional file 1: Table S4). At PN30, VGLUT1 showed equal staining intensity in the bipolar cell terminals of the OFF and ON sublamina in WT (Fig. 8e and Additional file 1: Table S4). In contrast, VGLUT1 staining was less prominent in the OFF sublamina than in the ON lamina of APLP2-KO (Fig. 8e and Additional file 1: Table S4), consistent with poor cone OFF differentiation showed above. Overall, these data showed that deletion of APLP2 was associated with altered development of bipolar cell terminals in the IPL suggesting APLP2 expression is necessary for the correct generation of the ON and OFF pathways.

Activation of microglial cells occurs during postnatal development to phagocytose inappropriate synapses to form a regular array in the two retinal plexiform layers [68]. Given the altered OPL and IPL in APLP2-KO, we hypothesized a change in microglial activation in these mice. WT retinas displayed no F4/80 immunostaining within the ONL, and rare immunostaining in the inner retinal layers. In contrast, processes of activated microglial cells were observed in the two plexiform layers and rarely in the INL of APLP2-KO (Additional file 1: Figure S6A). Classical complement cascade proteins have emerged as critical mediators of synaptic refinement. C1q refines the developing visual system through C3-dependent microglial phagocytosis of synapses [69]. We hypothesized alteration of the expression levels of the complement cascade in APLP2-KO. qRT-PCR data analysis showed C1qa, C1qb and C3 were significantly upregulated (by 45.3, 33.0 and 177.5 fold respectively) in the retina of APLP2-KO compared with WT, indicating overactivation of the complement cascade in adult APLP2-KO (Additional file 1: Figure S6B).

### Altered transcription of specific pre- and postsynaptic proteins in APLP2-KO

**Fig. 9:**
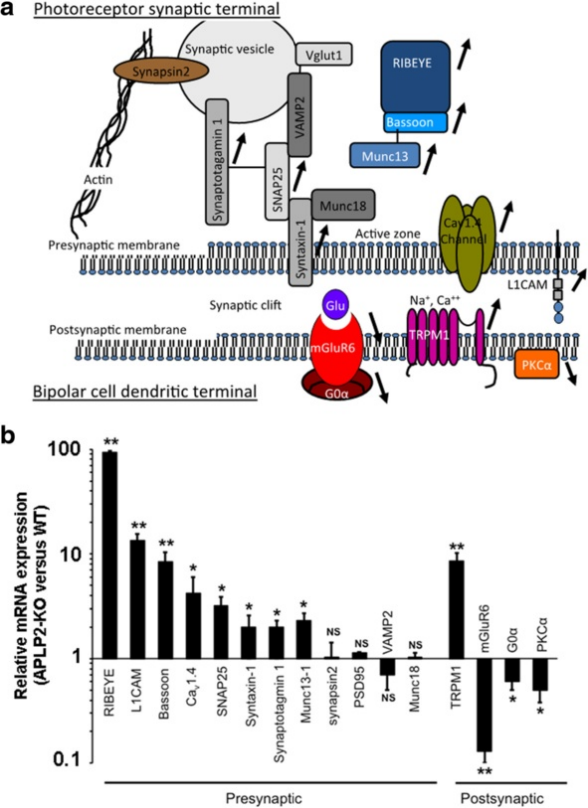
Altered transcription of specific complement pathway and pre- and postsynaptic proteins in APLP2-KO retina. **a** Schematic indicating the transcriptional alterations occurring in the pre- and postsynaptic gene network in the APLP2-KO retina. OPL synapse impairment was accompanied by specific change in synaptic gene expression showing enrichment (**↑**) of genes important for presynaptic function/organization ─ RIBEYE, Bassoon, Munc13, Syntaxin1, SNAP25, Synaptogamin-1, Cav1.4, L1CAM, TRPM1, and impoverishment (**↓**) of postsynaptic genes ─ mGluR6, Gα0 and PKCα. **b** Relative mRNA expression levels of differentially expressed genes determined by qRT-PCR and the calculated fold change in APLP2-KO retina relative WT. Mean ± s.e.m. *N* = 10 mice per genotype. *, *p* < 0.05; **, *p* < 0.01 vs WT (*t-test*)

Although CSNB is a synapse pathology, the transcriptional consequences on genes encoding pre- and postsynaptic proteins of gene mutations responsible of CSNB remain unknown. Therefore, we hypothesized that deleting APLP2 may affect a transcriptional network essential for retinal synapse structure/function. To gain insights into the molecular mechanisms underlying synaptic defects in APLP2-KO, we analyzed mRNA expression of 12 proteins specifically involved in synaptic organization and function (Fig. 9a). In adult APLP2-KO retinas, 8 of the 12 genes coding for presynaptic proteins were significantly upregulated (Fig. 9b): RIBEYE (93-fold, major component of the photoreceptor ribbon synapses); L1CAM (13-fold, necessary to establish precise synaptic contacts; bassoon (10-fold, main scaffold protein of the active zone that regulates stability of Munc13-1 and SNAP25); active zone-associated calcium channel Ca_v_1.4 (4-fold, necessary for maintaining bassoon in the ribbon synapse). Two main components of the soluble NSF attachment protein receptors, SNAP25 and syntaxin-1, were also upregulated. As were genes coding for two principal calcium sensors on synaptic vesicles, synaptotagmin 1 and Munc13-1 (2- to 3-fold). Genes coding for the presynaptic proteins: synapsin 2, PSD-95, synaptobrevin 2/VAMP2 and Munc18 had similar expression levels between WT and APLP2-KO.

In ON-bipolar cells, transmission of the light signal is mediated by G_O_α. In the dark, mGluR6 is constantly stimulated by glutamate and activates Goα which closes the TRPM1 channel, and PKCα accelerates and terminates glutamate-driven signal transduction. With the exception of TRPM1, which was significantly upregulated (9-fold), the other components of the ON bipolar cell signalling including mGluR6, G_O_α and PKCα were all significantly down regulated in APLP2-KO (Fig. 9b).

## Discussion

### Non-redundant roles for APLP2 and APP in the retina

The changes seen in synapse structure in the APLP2-KO retina are novel and are in contrast to the absence of synapse alterations in the APLP2-KO parasympathetic submandibular ganglion in the salivary duct [70], and the APLP2-KO neuromuscular junction in the diaphragm and sternomastoid muscles [71]. Sequence and phylogenetic analysis suggests there is functional conservation between APP and APLP1, but APLP2 having significantly diverged from both APP and APLP1 [5, 72]. Unlike other studies demonstrating that loss of APLP2 results in no gross abnormalities in the brain or PNS, we provide the first clear demonstration that APLP2 induces a profound phenotypic difference from APP. Our data have clearly identified non-redundant functions between APLP2 and APP in the adult retina and would indicate a specific role for APLP2 in retinal synaptogenesis and synaptopathy. Our data are consistent with recent data showing distinct and essential role of APLP2 at neuromuscular junction synapses that cannot be compensated by APP [73].

The exact molecular mechanism by which APLP2 is essential for synaps*e* formation and function in the CNS remains to be determined. One could formulate several hypotheses regarding how APLP2 affects retinal synaptogenesis and the transmission of the light signal from the photoreceptors to the bipolar cells. APP/APLP has been identified as a novel class of synaptic adhesion molecules with shared properties as SynCAMs and neuroligins/neurexins [3]. Roles of adhesion in the development and function of photoreceptor synapses remain incompletely understood. Unlike neuroligins [74, 75] or other immunoglobulin-family proteins, such as DSCAMs [76], that contribute to the development and function of the IPL, SynCAM 1 in the retina appears predominantly to provide for the structural and functional integrity of photoreceptor synapses in the OPL. SynCAM 1 is expressed on mouse rod photoreceptors and their terminals in the OPL in a developmentally regulated manner [77]. In adult, SynCAM 1 is expressed in OPL, where it encircles VGLUT1-positive photoreceptor terminals. Interestingly, the structural integrity of synapses in the OPL is impaired in SynCAM 1-KO mice [77]. Moreover, the number of ribbon-containing rod terminals was significantly lower and ribbons were shorter terminals with triads in SynCAM 1-KO mice, resembling retinal phenotype of APLP2-KO mice. Moreover, visual signal transmission in the scotopic (rod) pathway is impaired in SynCAM 1-KO mice. Consequently, APLP2 might play a SynCAM1-like synaptic adhesion function for OPL synaptogenesis and function. Besides a direct role in retinal synaptic adhesion, APP/APLP has been shown to interact with contactin 4 in the retina to promote NgCAM-dependent axon growth of ganglion cells in the chick [78]. Moreover, a subset of direction-selective ganglion cells fail to target the nucleus of the optic tract in APP-KO or contactin 4-KO mice [79]. Although these studies were limited to eye-to-brain connections, interactions of APLP2 with synaptic/cell adhesion molecules, including contactins, might be a mechanism by which APLP2 participates in OPL synaptogenesis, consistent with the identification of contactin 4 as a genetic marker of murine bipolar cells [80]. The third hypothesis is that APLP2 might directly or indirectly affect expression of other proteins. Unfortunately, no transcriptome profiling of retina in knockout mice lacking APP family members is available. However, the comparative transcriptome profiling of APP family members in adult cortex revealed that of 1061 genes that are altered after deletion of APLP2, only 17 % are also changed in APP-KO [81]. Moreover, brain interactomes for APLP2 and APP are different [82]. These studies suggest there are distinct pathways regulated by either APP or APLP2, consistent with the different APP and APLP2 retinal phenotypes. Among the nine APLP2-specific interactors identified in the brain [82], four are of potential interest because of their involvement in retinal development or retinal disorder: Retinitis Pigmentosa 2 homolog (RP2h), Ras-related C3 botulinum substrate 1 (RAC1), Protein phosphatase 2A (PP2A) and Rho family GTPase (RhoA). Mutation in the human RP2 gene cause X-linked retinitis pigmentosa [83, 84]. The Rp2h-KO mice showed significant reductions of the a-wave and b-wave amplitudes at 1 month of age and continued to decline over the next 6 months and exhibited a slowly progressing rod-cone dystrophy by 8 months [85]. In Rac1-KO mice, the numbers of cells in the ganglion cell layer and the INL are reduced to 50 % of the WT level, but the ONL is largely unaffected [86], implying a central role for Rac1in inner retinal development. PP2A dephosphorylates CaBP4 in the mouse retina [87]. Mutations in *CABP4* lead to iCSNB and mice lacking either Cabp4 display a iCSNB-like phenotype [38]. CaBP4 directly associates with the Cav1.4α channel. Increased CaBP4 phosphorylation potentiates the modulatory effect of CaBP4 on Cav1.3 Ca(2+) channels. RhoA signaling maintains apico-basal polarity in retinal progenitor cells, which is essential for subsequent cellular differentiation [88]. No retinal pathology has been described with RhoA dysfunction, so far.

### Molecular mechanisms underlying synaptic defects in APLP2-KO and animal models of CSNB

Although for different genes the pathogenic mechanisms of CSNB has been identified, for others further studies are needed to clarify the molecular mechanisms of CSNB. Loss-of-function mutations in some synaptic genes are responsible for alteration of the neurotransmission from photoreceptors to bipolar cells in iCSNB [35]. APLP2 localizes at both the dendrites of the bipolar cells and the presynatic terminals of the photoreceptors. Moreover, histology, immunohistochemistry and TEM of APLP2-KO retinas showed alterations on both the presynaptic terminals of rods and cones, and the dendritic tips of the bipolar cells, making APLP2-KO a pathology of both the pre- and postsynatic part of the OPL.

Analysis of the expression of pre- and postsynatic genes in CSNB and animal models of CSNB has been rarely performed to identify the molecular mechanism involved or associated with the pathogenic events in CSNB. The transcriptome analysis of the genes coding pre- and postsynaptic proteins in APLP2-KO retinas, indicates enrichment of genes important for presynaptic function/organization ─ RIBEYE, Bassoon, Munc13, Syntaxin1, SNAP25, Synaptogamin-1, Cav1.4, L1CAM, TRPM1, and impoverishment of postsynaptic genes ─ mGluR6, Gα0 and PKCα (summarized in the schema, Fig. 9a). Deletion of APLP2 also results in a severe reduction of mGluR6 at the dendritic tips of rod bipolar cells. Therefore, our study showed a reduction in the expression of major components of the mGluR6 signaling cascade in APLP2-KO. Interestingly, GRM6- and NYX- and LRIT3 deficient mouse, three models for cCSNB, showed a nearly absent mGluR6 at the dendritic tips of cone ON-bipolar cells [71, 89–91], indicating a reduction in ON-bipolar cells of major components of the mGluR6 signaling cascade in mouse models of cCSNB. By RT-qPCR, we demonstrated that APLP2 deletion alters the expression of *TRPM1*, *GRM6* and *CACNA1F*, three major genes involved in CSNB. This suggests that even in the absence of any mutation, APLP2 may be involved in the phenotype of CSNB by its regulation function of expression of genes directly involved in this pathology. Recently, a new naturally occurring animal model of cCSNB has been characterized in beagle dog [47]. All known CSNB genes have been excluded in this model. However, a reduction in the expression of the photoreceptor postsynaptic gene *PKCA* and an increased expression of the photoreceptor postsynaptic gene TRPM1 were observed in this model, as we observed in APLP2-KO mice. It remains to investigate the expression of APLP2 in this model.

### New roles for retinal differentiation genes in CSNB?

APLP2 deletion alters the expression of bHLH and HD transcription factors that are involved in the generation and differentiation of bipolar cells at early and late postnatal stages of development. Since CSNB is present at birth and ERG b-wave is reduced in mice either lacking Bhlhb4 or Math5, two transcription factors required for maturation/differentiation of bipolar cells, it was suggested that altered differentiation of bipolar cells may be involved in the phenotype of CSNB [44, 45]. Recently, the differentiation factor, PRDM8, was presented as a candidate gene for CSNB [46], reinforcing the importance to study the mechanisms of bipolar cell differentiation for elucidating the pathological events involved in CSNB. ON-bipolar (G0α-positve cells), rod bipolar (PKC-positive cells) and type 2 (recoverin-positive cells) cone bipolar cells were markedly reduced in PRDM8-KO mice [46]. Reduced VGLUT1 staining was also observed in the IPL of PRDM8-KO mice. Moreover, a reduced scotopic b-wave amplitude with a normal a-wave amplitude was observed in PRDM8-KO mice [46]. The ERG deficits of PRDM8-KO mice were stationary between 3 and 6 months of age. The stationary ERG phenotype of APLP2-KO mice was observed between 2–4 months and 6–13 months of age. An additional role of APLP2 in the retina is the control of bipolar cell (ON-bipolar, rod bipolar and type 2 cone bipolar cells) differentiation, development of bipolar cell dendrites, temporal sequence of VGLUT1 expression from OFF to ON laminae during the differentiation of the IPL and the formation of cone and rod ribbon synapses during postnatal development. Therefore, we cannot rule out that APLP2 may also be involved in CSNB phenotype by its function of differentiation factor. This is in accordance with the recently suggested role of LRIT3 in cone synapse formation [89], confirming the importance of photoreceptor synaptogenesis in CSNB. However, alteration of ribbon synapse formation or bipolar dendrite invagination has been described in GRM6 mutants and Gβ5-KO mice, two models of cCSNB [92–95]. Moreover, Ca_v_1.4 and CaBP4-KO mice exhibit altered photoreceptor synaptogenesis [42], confirming that impaired retinal differentiation contributes to vision impairment in CSNB. It remains to be determined if APLP2 expression is altered in the bipolar cells and photoreceptor ribbon synapses of GRM6-, TRPM1-, NYX-, GRP179-, Ca_v_1.4-, CaBP4- or LRIT3-KO mice during development and in adults.

## Methods

### Animals

The homozygous APP and APLP2 knockout and wild-type (C57/Bl6) mice have been described [96–98]. Eye balls were harvested from mice at different ages ranging from embryonic day (E) 14 and E18, and postnatal day (PN) 1, 5, 10, and 15, and from young (2–4 months) and adult (6–13 months). All Procedures were approved by the Animal Ethics Committee at the Pierre et Marie University and Paris-Descartes University of Paris and were carried out in accordance with French laws and the European Union Directive 2010/63/EU.

### SD-OCT

In vivo assessment of mice retinal thickness was performed on anesthetized animals using spectral domain optical coherence tomography (SD-OCT) (Spectralis, Heidelberg Engineering) adapted for small animal eyes. Temporal, nasal, and superior quadrants of the retina were analyzed, using the optic nerve head and the retina vessels as landmarks. Each 2-dimensional B-scan recorded at 30° field of view consisted of 1536 A-scans with an optical resolution reaching 3.5 μm. Thickness of full retina, inner retina, and ONL were quantified using software provided by Heidelberg Engineering (Heidelberg Eye Explorer Version 1.6.2.0).

### Measurement of retinal thickness

Retina cross-sections were mounted and stained with toluidine blue. Images of whole retina cross-sections were obtained using Leica leitz Aristoplan microscope equipped with DFC 480 camera (Leica) and LAS 3.7 version software. Thickness of the different nuclear and synaptic layers were determined using Visilog 6.4 version software (Noesis).

### Immunohistochemistry

Retinal expression of APLP2 during embryonic and postnatal development was investigated by immunohistochemistry as previously described [18]. Serial eye sections were stained with primary rabbit anti-APLP2 antibody and detected with a secondary antibody, a goat polyclonal biotin-conjugated anti-rabbit IgG (1/1000, Vector Laboratories) and visualized using streptavidin and biotinylated horseradish peroxidase complex (sABC) and diaminobenzidine tetrahydrochloride (DAB). The peroxidase-stained sections were viewed with an Aristoplan-microscope (Zeiss) connected to a digital camera.

### Immunochemistry

Identification and visualization of specific cells and cellular structures in the retina was performed by immunofluorescence microscopy as previously described [18]. Primary antibodies are shown in Additional file 1: Table S1. Control experiments to check specificity of the primary antibodies: APLP2 antibody was omitted and secondary antibody was tested. Retinas of at least three animals were used at each stage. Nuclei were counterstained with DAPI (1:4000, Sigma-Aldrich). Sections were mounted with Dako (Cytomation). Slides were observed with a Zeiss confocal Imaging system (LSM710, Zeiss). All immunostaining was repeated at least three times. To quantify the number of different cell-type markers on retinal sections, three or more age-matched retinas were analyzed for each cell type. At least five non-overlapping fields of 400 μm of linear retinal length were photographed and printed at 40X magnification. They were counted from each retina section (4 or more sections per animal) and averaged per group.

### Transmission electron microscopy

After dissection, retinas were fixed in 2.5 % glutaraldehyde cacodylate buffer (0.1 M, pH 7.4) and then fixed in 1 % osmium tetroxyde in cacodylate buffer (0.2 M, pH 7.4) and progressively dehydrated in graduated ethanol solution, then in propylene oxide. Semi-thin sections (1 μm) were cut with an ultra microtome (Reichert OmU2) and stained with toluidine blue. Ultra-thin sections (80 nm) were contrasted by uranyl acetate, and analyzed with a transmission electron microscope (Philips CM10).

### Western blot analysis

Retinas were lysed in ice-cold lysis buffer (50 mM Tris–HCl, pH 7.5, 100 mM NaCl, 0.1 % NP-40, 1 % deoxycholate, 50 mM β-glycerophosphate, 0.2 mM sodium orthovanadate, 50 mM sodium fluoride, 1 μg/mL leupeptin, 5 μM pepstatin, 20 kIU/mL aprotinin, 1 mM phenylmethylsulfonyl fluoride), and centrifuged for 10 min at 10,000 *g* at 4 °C. Protein concentrations were determined using a Bio-Rad kit. Retina lysates were mixed with 3× Laemmli buffer and heated for 5 min at 95 °C. They were then resolved by SDS-PAGE (10 % or 15 % polyacrylamide gels), electroblotted onto polyvinylidene difluoride (PVDF) membrane (Immobilon; Millipore), and probed with antibodies directed against Chx10, Islet1, VGLUT1, RIBEYE mGluR6 and actin (Additional file 1: Table S1). Primary antibodies were tagged with specific secondary horseradish peroxidase-conjugated antibodies. Antibody complexes were detected by enhanced chemiluminescence (ECL; Amersham), and the membrane was placed against Kodak film (BioMax Light-1; Eastman Kodak). Quantification was carried out using a Kodak image station (2000 MM) and Kodak software (1D3.6).

### Quantitative real-time PCR

Mouse retina tissue was exposed to TRIZOL reagent (Invitrogen, France) according to the manufacturer’s instructions, and SuperScript II Reverse Transcriptase (Invitrogen) was used to reverse transcribe 1 μg of mRNA. Amplification reaction assays contained 1× SYBR Green PCR Mastermix (Applied Biosystems). All real-time PCR oligonucleotide primers had previously been validated experimentally by qPCR, agarose gel analysis, sequencing, and blast. The PCR primer sequences are available on request. A hot start at 95 °C for 5 min followed by 40 cycles at 95 °C for 15 s and 60 °C for 1 min with the 7300 SDS thermal cycler (Applied Biosystems). Controls with no reverse transcriptase were run for each assay to confirm the absence of genomic DNA contamination. Control qRT-PCRs were performed without cDNA templates. The standard curve method (Prism 7700 Sequence Detection System, Applied Biosystems, ABI User Bulletin number 2) was used for relative quantification of gene expression. For each experiment, each individual sample was run in triplicate and the *C*_t_ value for each sample recorded at the end of the reaction. The average and standard deviation of the three *C*_t_ values were calculated. Gene expression levels were normalized to β-actin for each retinal tissue sample and calculated relative to WT retinal tissue with the following equation: relative expression = 2^─(APLP2 sample Δ*C*t ─ WT sample Δ*C*t)^ where Δ*C*_t_ = mean *C*_t_(target) − mean *C*_t_(β-actin).

### Electroretinography

Animals were dark adapted overnight. Under dim-red-light, they were anesthetized with a mixture of ketamine/xylazine and their pupils dilated with 1 drop of atropine. A photostimulator (Type PS 33) was used to generate ERG. The highest flash stimulation was 11.8 cd.s.m^2^ and neutral density filter were used to attenuate luminance. Flash stimuli were given through an integrative sphere in order to uniformly illuminate all retina. A single ERG was recorded for seventeen increasing luminance (10 μs duration, 30s apart) via Ag/AgCl electrodes. The signal was amplified (gain 1000, pass band 0.1-10,000 Hz; A-M systems, Inc; Model 3000. AC/DC Differential Amplifier) then processed and stored in a computer. For each ERG, a- and b-wave amplitude and latency were calculated. The mean values are plotted against the flash stimulation luminance. The software program (Microsoft Origin 6.0; Microcal Software) was used to fit the b-wave sensitivity curves to calculate the saturated b-wave amplitude (*B*_max_), the half-saturation luminance (*K*); and the slope of the curve in its linear part (n). For photopic conditions, mice were light-adapted 10 min at 30 cd/s m^2^ and then the retinal activity recorded in response to single flashes ranging from 10^−2^ to 30 cds/m^2^, in the same illumination conditions. For each stimulus intensity, 10 to 15 traces were averaged. Amplitude of the a-wave was defined as the difference between the baseline level at the time of stimulation and the peak of the a-wave. Amplitude of the b-wave was defined as the difference between the peak of the b-wave and the peak of the a-wave (or the baseline level when the a-wave was not detectable). Amplitudes are expressed in μV.

### Statistical analysis

All data are presented as mean ± s.e.m. Statistical significance in pairwise comparisons was assessed using two-tailed t-tests with unequal variance.

## Conclusions

In conclusion, our findings demonstrated the important and specific role of APLP2 in the retina. Our results demonstrate the non-redundant actions of APLP2 and APP in the central nervous system that are mediated through different effects on specific neurons and on ribbon synaptic networks during late embryonic and early postnatal development. Most importantly and of clinical relevance, there are striking similarities to be found between the phenotypic features of bipolar cell dendrites and photoreceptor ribbon synapses, and vision alteration in APLP2-KO mice and congenital stationary night blindness. APLP2 may be a bridge between retina development and synaptic genes involved in the pathogenic events in CSNB. Our studies have identified APLP2 as a new molecule that warrants investigation for its involvement in a range of eye pathologies.

## Abbreviations

APLP2, amyloid precursor-like protein 2; APP, amyloid precursor protein; BHLH, basic helix-loop-helix; BHLHB4, basic helix-loop-helix domain containing, class B 4; C1q, complement 1 q; CABP4, calcium binding protein 4; CACNA1F/Ca_v_1.4, calcium channel, voltage-dependent, L type, alpha 1 F subunit; cGMP, cyclic guanosine monophosphate; Chx10, Ceh-10 homeodomain-containing homolog; CNS, central nervous system; CSNB, congenital stationary night blindness; DSCAM, down syndrome cell adhesion molecule; ERG, electroretinogram; GCL, ganglion cell layer; Gγ13, G protein gamma 13 subunit; HD, homeodomain; INL, inner nuclear layer; IPL, inner plexiform layer; IS, inner segment; Islet1, insulin related protein 1; L1CAM, L1 cell adhesion molecule; LRIT3, Leucine-rich repeat, immunoglobulin-like and transmembrane domains 3; MATH5/ATOH7, atonal BHLH transcription factor 7; mGluR6, metabotropic glutamate receptor 6; mRNA, messenger ribonucleic acid; NBL, neuroblastic layer; NFL, nerve fiber layer; NYX, nyctalopin; OCT, optical coherence tomography; OLM, outer limiting membrane; ONL, outer nuclear layer; OPL, outer plexiform layer; OS, outer segment; OTX2, orthodenticle homeobox 2; PKCα, protein kinase C alpha; PN, postnatal day; PNA, peanut agglutinin; PNS, peripheral nervous system; PP2A, Protein phosphatase 2A; PRDM8, RIZ homology domain containing 8; PSD95, postsynaptic density protein 95; RAC1, Ras-related C3 botulinum substrate 1; RGC, retinal ganglion cells; RhoA, Rho family GTPase; RP2h, Retinitis Pigmentosa 2 homolog; RPE, retinal pigment epithelium; RT-PCR, reverse transcription polymerase chain reaction; SD-OCT, spectral domain optical coherence tomography; SNAP25, synaptosomal-associated protein 25; SynCAM, synaptic cell adhesion molecule; TEM, transmission electron microscopy; TRPM1, transient receptor potential cation channel subfamily M member 1; VGLUT1, vesicular glutamate transporter 1; VSX1, visual system homeobox 1; WT, wild-type; XRLS, X-linked Retinoschisis

## Additional file

Additional file 1: Figure S1. APLP2 expression was localized in the two synaptic layers and overlapped extensively with APP in specific neuronal populations of the adult retina. Figure S2. The ultrastructure of the RPE in the adult retina did not differ between WT and APLP2-KO. Figure S3. Impaired retinal function at the level of the post-rod response in APLP2-KO. Figure S4. The ultrastructure of the ribbon photoreceptor presynaptic terminals differed dramatically between young WT and APLP2-KO. Figure S5. Deletion of APLP2 delays the development of the OPL. Figure S6. Perturbed retinal microglia cell activation and altered transcription of specific complement pathway in APLP2-KO. Table S1. Antibodies used for colabeling immunofluorescence experiments. Table S2. Loss of APLP2 does not alter gross lamination of young and adult retina. Table S3. APLP2 is required for the differentiation of bipolar and synaptogenesis of cone and rod terminals during postnatal development. Table S4. APLP2 is necessary for the generation of the ON and OFF pathways through the proper development of bipolar cell terminals during postnatal development. (DOC 4053 kb)
