# Supplementary material for: Amyloid Precursor-Like Protein 2 deletion-induced retinal synaptopathy related to congenital stationary night blindness: structural, functional and molecular characteristics
Source: Mol Brain. 2016 Jun 8;9:64. doi: 10.1186/s13041-016-0245-z (PMC4897877; doi:10.1186/s13041-016-0245-z)
Supplement: Additional file 1: Figure S1. — APLP2 expression was localized in the two synaptic layers and overlapped extensively with APP in specific neuronal populations of the adult retina. Figure S2. The ultrastructure of the RPE in the adult retina did not differ between WT and APLP2-KO. Figure S3. Impaired retinal function at the level of the post-rod response in APLP2-KO. Figure S4. The ultrastructure of the ribbon photoreceptor presynaptic terminals differed dramatically between young WT and APLP2-KO. Figure S5. Deletion of APLP2 delays the development of the OPL. Figure S6. Perturbed retinal microglia cell activation and altered transcription of specific complement pathway in APLP2-KO. Table S1. Antibodies used for colabeling immunofluorescence experiments. Table S2. Loss of APLP2 does not alter gross lamination of young and adult retina. Table S3. APLP2 is required for the differentiation of bipolar and synaptogenesis of cone and rod terminals during postnatal development. Table S4. APLP2 is necessary for the generation of the ON and OFF pathways through the proper development of bipolar cell terminals during postnatal development. (DOC 4053 kb) [file 13041_2016_245_MOESM1_ESM.doc]

**Amyloid Precursor-Like Protein 2 deletion-induced retinal synaptopathy related to congenital stationary night blindness**: s**tructural, functional and molecular characteristics**

Virginie Dinet^1^, Giuseppe D. Ciccotosto^2^, Kimberley Delaunay^1^, Céline Borras^1^, Isabelle Ranchon-Cole^3^, Corinne kostic^4^, Michèle Savoldelli^1^, Mohamed El Sanharawi^1^, Laurent Jonet^1^, Caroline Pirou^1^, Na An^1^, Marc Abitbol^1^, Yvan Arsenijevic^4^, Francine Behar-Cohen^1^, Roberto Cappai^2^, and Frédéric Mascarelli^1^

(1) Centre de Recherche des Cordeliers, Université Paris Descartes, Université Pierre et Marie Curie, Paris, France, (2) Department of Pathology and Bio21 Molecular Science and Biotechnology Institute, The University of Melbourne, Melbourne, Australia, (3) Laboratoire de Biophysique Sensorielle, Université Clermont 1, Clermont-Ferrand, France, (4) Unit of Gene Therapy & Stem Cell Biology, University of Lausanne, Jules-Gonin Eye Hospital, Lausanne, Switzerland.

Corresponding author: Frédéric Mascarelli. frederic.mascarelli@inserm.fr

**Additional file 1**

**Fig. S1. APLP2 expression was localized in the two synaptic layers and overlapped extensively with APP in specific neuronal populations of the adult retina.** The APLP2-positive cells were determined by co-immunostaining with (a-f) anti-APLP2 (green) and cell specific markers for (A) anti-Pax6 (red), (B) anti-glycine receptor (red), (C) anti-calretinin (red), (D) anti-ChAT (red), (E) anti-PKCα (red), and (F) anti-calbindin (red). Coexpression was visualized by the orange to yellow staining pattern. (A) Using anti-Pax6 antibody, we showed APLP2 colocalized with RGCs (arrowheads) and amacrine cells (arrows) while the (B) anti-glycine receptor specifically labeled glycinergic RGCs (arrowheads), AII amacrine cells (white arrows) and bipolar cells (black arrows). (C) Coimmunostaining with calretinin confirmed APLP2 expression in amacrine cells (arrows) and ganglions cells (arrowheads). Moreover, calretinin labeled three bands in the IPL, the upper and lower ones corresponding to the OFF and ON starburst amacrine cell plexuses, respectively (C). (D) Anti-ChAT antibody showed APLP2 colocalized with cholinergic (starburst) amacrine cells (arrows) and the two stratified bands (S2 and S4) of the cholinergic synapses in the IPL and confirmed APLP2 in ganglion cell axons (arrowheads). (E) Co-staining with PKCα revealed APLP2 expression in the somas (arrows) and dendrites (arrowheads) of rod bipolar cells. In the OPL, APLP2 colocalized with VGLUT1 (F) and PSD95 (G) (arrowheads). (H) costaining for calbindin showed APLP2 was expressed in the somas (arrows) and terminals (arrowheads) of horizontal cells. Nuclei were counterstained with DAPI (blue). (I) A toluidine blue-stained semithin resin section revealed normal histology of WT adult retina. (J-L) Longitudinal cryostat sections of (J-L) WT and (M) APLP2-KO retinas immunostained with (J, L and M) anti-APLP2 and (K) anti-APP antibody. (L) Double immunostaining with anti-APLP2 and anti-APP antibodies showed that APLP2 colocalized with APP in all synaptic and nuclear layers of neural retina, with the exception of the outer segment (OS) of the photoreceptors (asterisk), indicating APLP2 expression, in large part, overlapped the laminar distribution of APP. (M) Immunostaining with anti-APLP2 antibody on APLP2-KO retinas produced no specific labeling in the neuronal cell layers from NFL to OS. Sections were analyzed by confocal laser scanning microscopy. Asterisk indicates OS. NFL: nerve fiber layer, CGL: ganglion cell layer, IPL: inner plexiform layer, INL: inner nuclear layer, OPL: outer plexiform layer, ONL: outer nuclear layer. OLM: outer limiting membrane, IS: inner segment, OS: outer segment. Scale bars (A-G. G-K) 50 µm and (H) 20 µm.

**
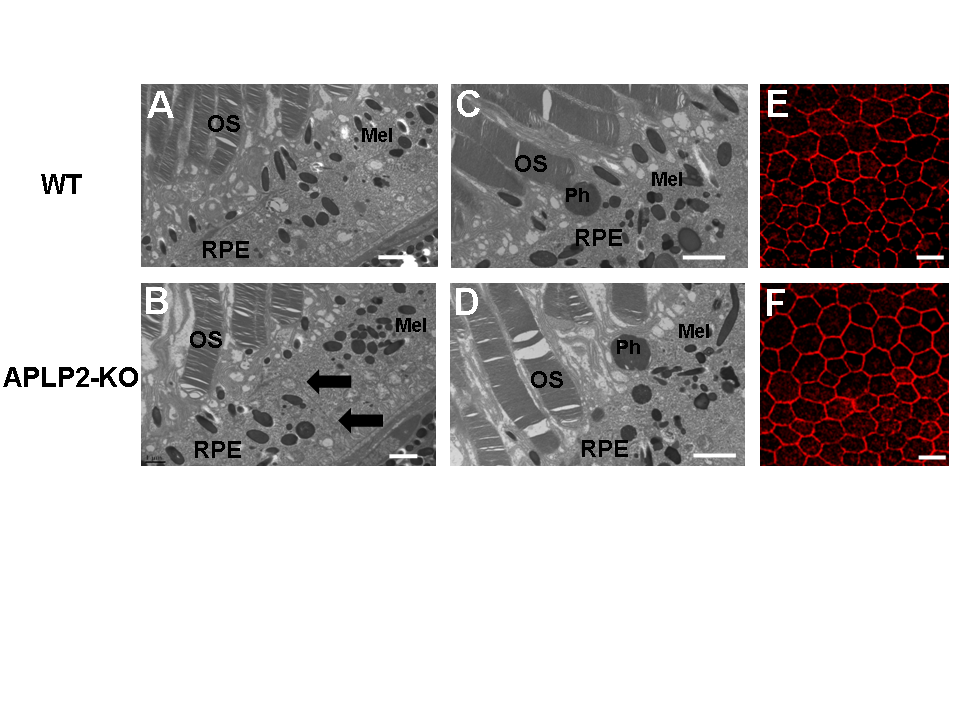
**

**Fig. S2 The ultrastructure of the RPE in the adult retina did not differ between WT and APLP2-KO.** (A-D) Electron micrographs of the RPE showed that WT (A and C) and APLP2-KO (B and D) mice presented a normally developed and regular RPE. The RPE apical microvilli ensheath the outer segments of photoreceptors (OS), indicating the absence of retinal detachment. In WT and APLP2-KO mice, RPE contained numerous elongated melanin granules (Mel). The RPE contained numerous phagosomes (Ph) near the infoldings of the basal plasma membrane. The RPE were without cytoplasmic vacuoles and presented a continuous junction complex between two adjacent cells (arrow). Scale bars: 2 µm. (E and F) RPE/choroid flat mounts were stained with phalloidin-RITC to examine the F-actin cytoskeleton of the RPE. RPE flat mounts were counterstained with DAPI to detect nuclei. WT (E) and APLP2-KO (F) mice showed a regular monolayer of well-juxtaposed hexagonal RPE cells.

**
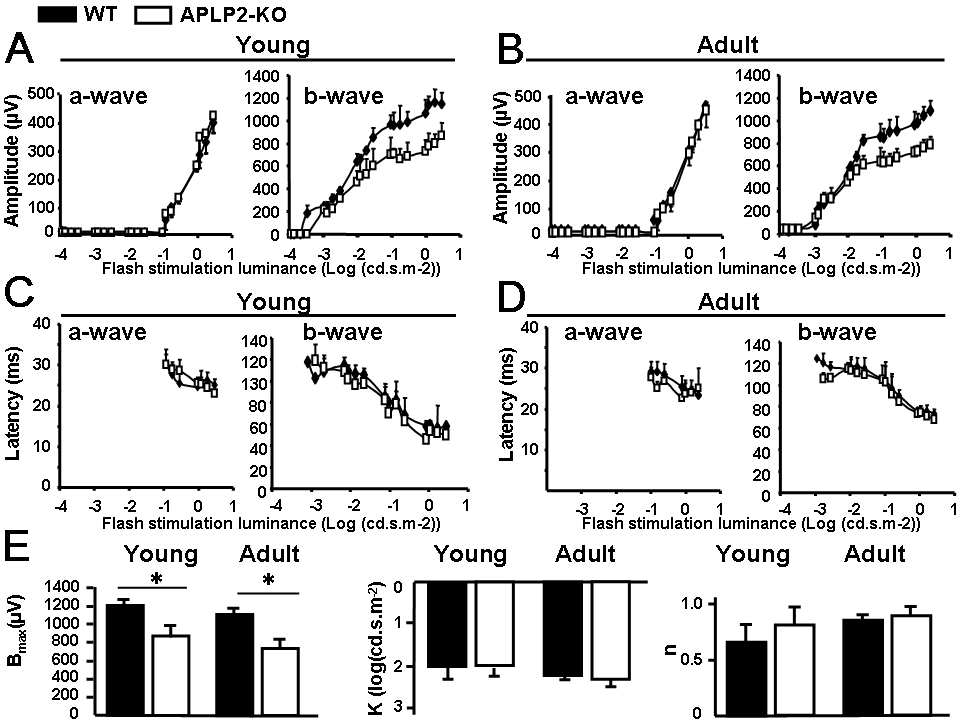
**

**Fig. S3**. **Impaired retinal function at the level of the post-rod response in APLP2-KO.** (A) Amplitude of a-wave and b-wave in young APLP2-KO and WT, (B) amplitude of a-wave and b-wave in adult APLP2-KO and WT, (C) latency of a-wave and b-wave in young APLP2-KO and WT and (D) latency of a-wave and b-wave in adult APLP2-KO and WT plotted as a function of the flash stimulation luminance. ERG analysis revealed a reduction of the b-wave amplitude in young and adult APLP2-KO mice compared to WT without alterations to a-wave amplitude and latency. The b-wave curve for each animal was fitted to calculate the derived parameters for (E) maximal b-wave amplitude (*B_max_*), the half saturation luminance (*K*) and the slope (*n*) in the linear part. Mean ± s.e.m. N= 7 mice per genotype. *, p<0.05 vs WT (*t-test*).

**
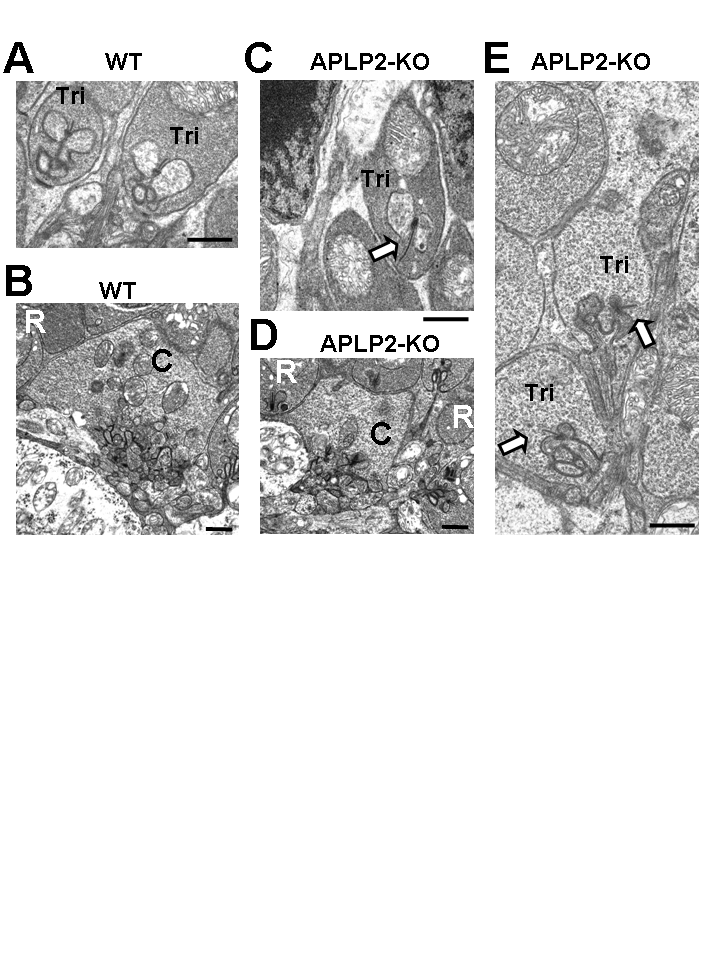
**

**Fig. S4 The ultrastructure of the ribbon photoreceptor presynaptic terminals differed dramatically between young WT and APLP2-KO**. Electron micrographs of longitudinal sections of (A and B) WT and (C-E) APLP2-KO. (A) In the young WT OPL, the rod spherules contained a well-formed triad composed of two horizontal cell lateral processes and a central, postsynaptic, bipolar cell terminal. Photoreceptor terminals showed an unique long presynaptic ribbon. (B) The cone pedicles of young WT showed a regular form. They contained multiple mitochondrion and triads composed of two lateral horizontal processes flanking a central bipolar cell dendrite. In APLP2-KO, rod spherules displayed ribbon synapses (C, arrow) facing the ONL or (E, arrows) laterally oriented, instead of facing the OPL. (D) cone pedicles were irregular in shape and completely disorganized in APLP2-KO. Cone terminal (C), rod terminal (R), Tri (triad). Scale bars: 1 µm.

**
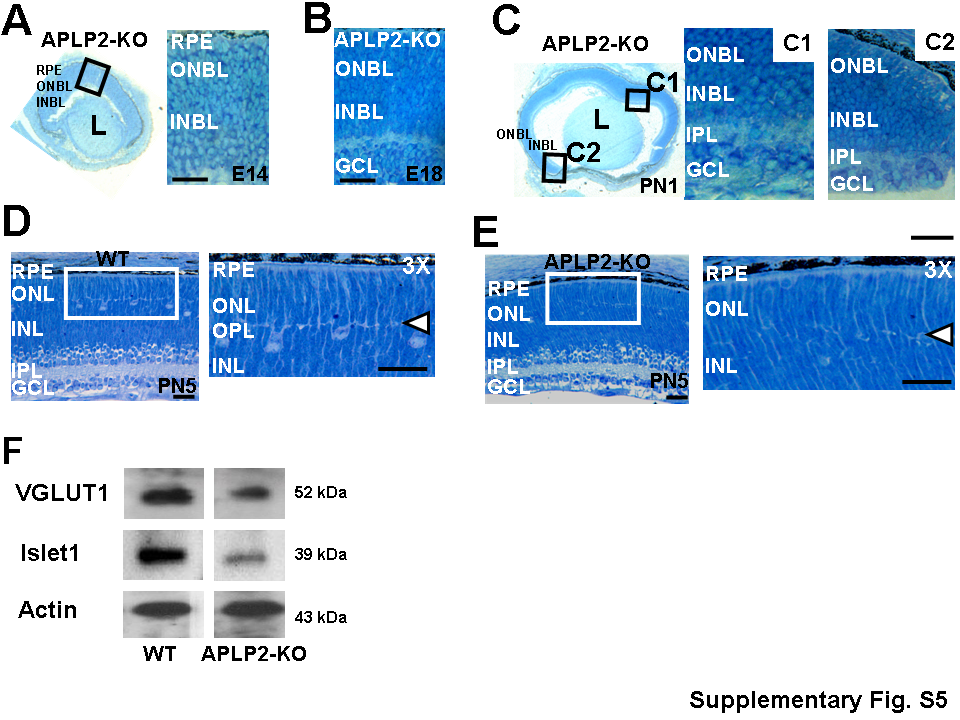
**

**Fig. S5. Deletion of APLP2 delays the development of the OPL.** Histology of 80 nm-thick resin retina sections showed normal laminar structure at developmental stages E14 (A), E18 (B) and PN1 (C). c1 and c2 show magnified region from PN1 central and peripheral APLP2-KO retina respectively. (D) At PN5, the OPL layer which was present in WT retina and highlighted (arrow) at higher magnification was not present in (e) APLP2-KO retina. Sc/Cho: sclera and choroid, Cho: choroid, ONBL: outer neuroblastic layer, INLB: inner neuroblastic layer, L: lens, OR: outer retina, IR: inner retina. Scale bars: 50 µm. Western blot analysis of WT and APLP2-KO protein extracts of the PN5 retinas were probed for VGLUT1 (62 kDa), Islet1 (39 kDa) and the loading control, actin (43 kDa).

**
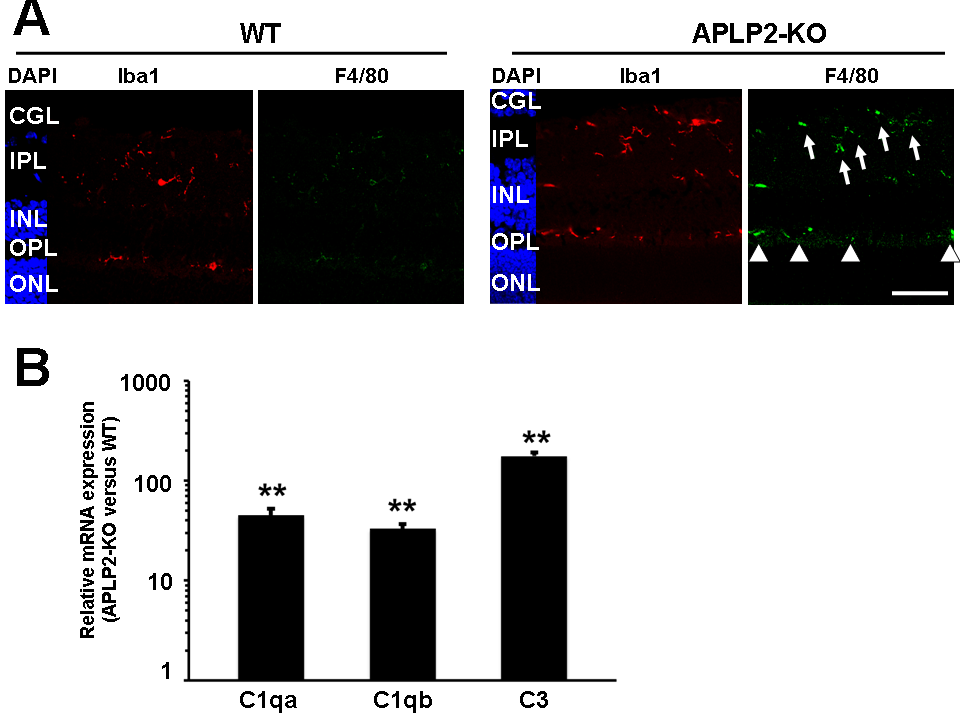
**

**Fig. S6. Perturbed retinal microglia cell activation and altered transcription of specific complement pathway in APLP2-KO**. (A) Longitudinal cryostat retina sections of adult WT and APLP2-KO underwent double immunostaining with anti-Iba1 and anti-F4/80 antibodies to identify the total population of microglial cells and activated microglial cells, respectively. DAPI (blue) was used to counterstain nuclei. Anti-Iba1 (red) and anti-F4/80 (green) immunofluorescence detected a different spatial localization and activation of microglial cells during development in APLP2-KO retinas when compared with WT. No microglial cells were activated in WT, whereas processes of activated microglial cells were still observed in the two plexiform layers (IPL: arrows, OPL: arrowheads) and rarely in the INL of APLP2-KO. (B) Relative mRNA expression levels of differentially expressed genes determined by qRT-PCR and the calculated fold change in APLP2-KO retina relative WT. Mean ± s.e.m. N= 10 mice per genotype. **, p< 0.01 vs WT (*t-test*). GCL ganglion cell layer, INL inner nuclear layer, I. INL inner portion of the inner nuclear layer, O.INL outer portion of the inner nuclear layer, ONL outer nuclear layer, IPL inner plexiform layer, OPL outer plexiform layer. Scale bars: 150 µm.

**
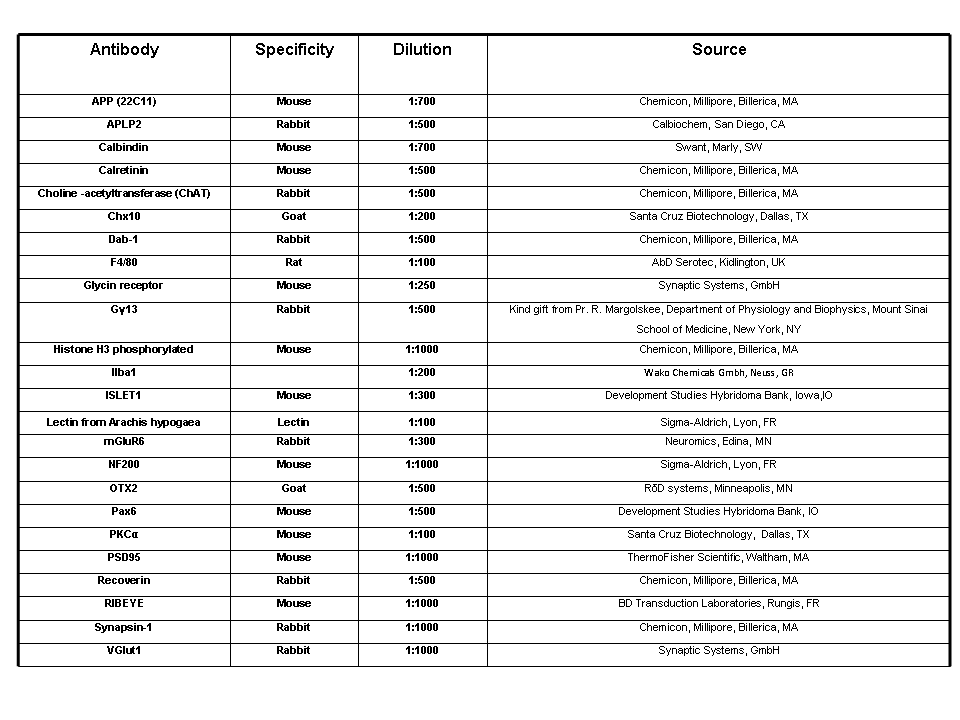
**

**Table S1. Antibodies used for colabeling immunofluorescence experiments.**

**
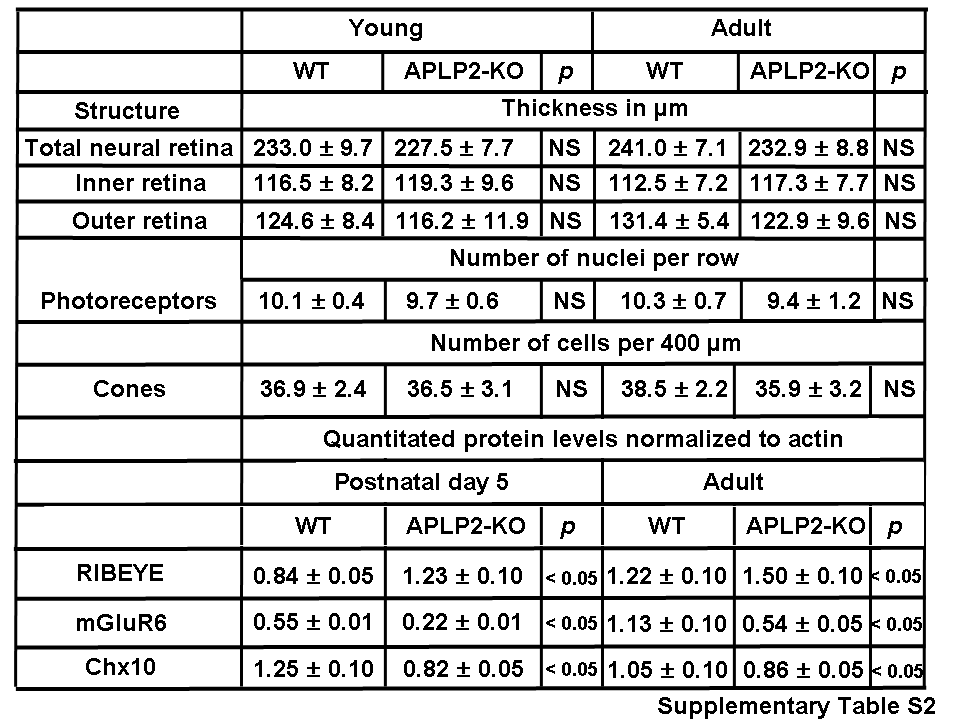
**

**Table S2. Loss of APLP2 does not alter gross lamination of young and adult retina**. *In vivo* assessment of mice retinal thickness was performed on anesthetized animals using SD-OCT. Thickness of full retina, inner retina, and ONL were quantified using software provided by Heidelberg Engineering. The number of rods and cones were similar between the two genotypes All data are presented as mean ± s.e.m. N= 4. *p* value; NS > 0.05. Quantitative analysis of the amounts of RIBEYE, mGluR6 and Chx10 from the Western blot of the PN5 and adult retinal extracts of APLP2-KO and WT mice. Each protein within each lane was quantified and normalized to actin within that lane. Data of the ratios are plotted for each protein and presented as mean ± s.e.m. N= 3. *p* value.

**
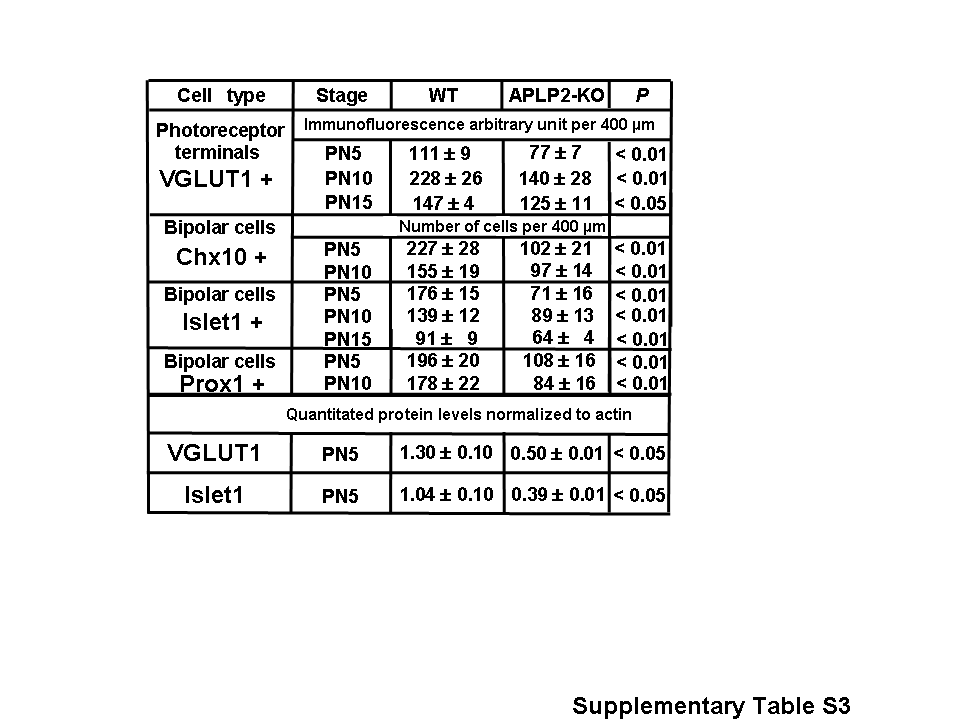
**

**Table S3.** **APLP2 is required for the differentiation of bipolar and synaptogenesis of cone and rod terminals** **during postnatal development.** Longitudinal cryostat sections of retinas from the indicated stages of postnatal development were immunostained for Chx10, Islet1 and prox1 was performed to identify the differentiation of bipolar cells. Number of marker positive cells was counted per 400 µm central retina. The marked decreased in Chx10-, Islet1- and prox1-positive cells through the middle portion of the INL in APLP2-KO compared to WT, indicates a major role of APLP2 in bipolar cell differentiation. Expression of VGLUT1 was analyzed to investigate temporal ordering in development of rod versus cone terminals in the OPL. Quantitative analysis of confocal images for VGLUT1 immunostaining showed that there was a significant delay in the development of photoreceptor presynatic terminals during postnatal retinal development in APLP2-KO as compared with WT. Immunostaining for All data are presented as mean ± s.e.m. N= 3. *p* value. Quantitative analysis of the amounts of VGLUT1 and Islet1 from the Western blot of the PN5 retinal extracts showed an overall marked decrease in the levels of the two proteins in APLP2-KO mice. Each protein within each lane was quantified and normalized to actin within that lane. Data of the ratios are plotted for each protein and presented as mean ± s.e.m. N= 3. *p* value.

**
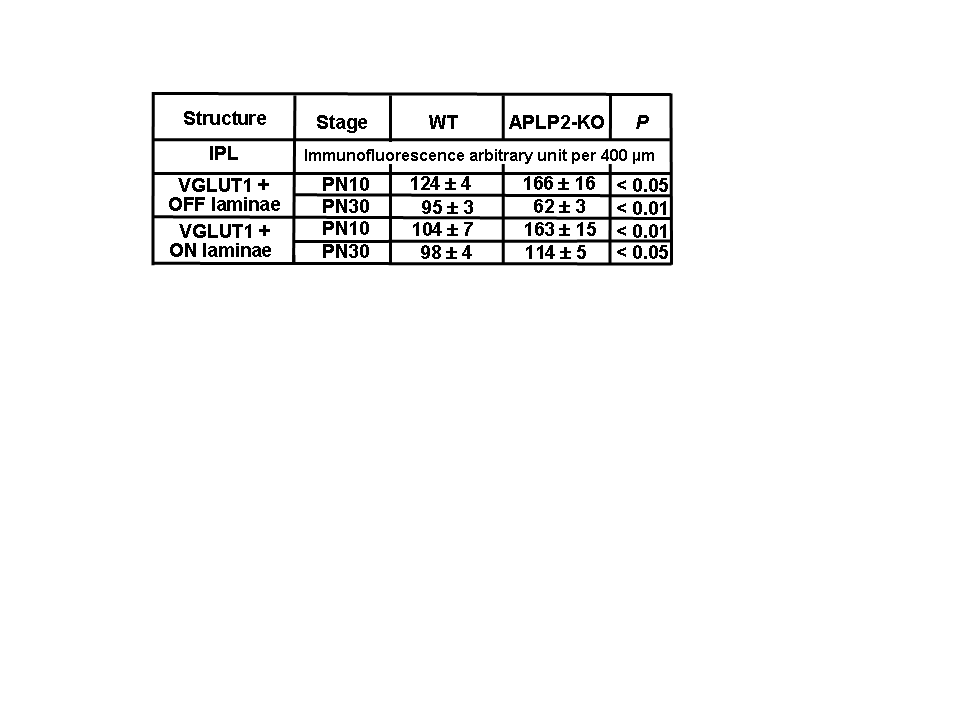
**

**Table S4.** **APLP2 is necessary for the generation of the ON and OFF pathways through the proper development of bipolar cell terminals during postnatal development.** Longitudinal cryostat sections of retinas from the indicated stages of postnatal development were immunostained for VGLUT1 to study the development of the laminar organization of the IPL. Quantitative analysis of confocal images for the VGLUT1 immunostaining was performed to investigate the temporal sequence of VGLUT1 expression in bipolar cell terminals from OFF to ON laminae during the differentiation of the IPL. The altered development of the VGLUT1 labeled bipolar cell terminals in the IPL of APLP2-KO compared to WT indicated that APLP2 is necessary for the proper generation of the ON and OFF pathways. All data are presented as mean ± s.e.m. N= 3. *p* value.
